# [Ag_115_S_34_(SCH_2_C_6_H_4_^*t*^Bu)_47_(dpph)_6_]: synthesis, crystal structure and NMR investigations of a soluble silver chalcogenide nanocluster†
†Dedicated to Evamarie Hey-Hawkins on the occasion of her 60^th^ birthday.
‡
‡Electronic supplementary information (ESI) available: CCDC 1507868. For ESI and crystallographic data in CIF or other electronic format see DOI: 10.1039/c6sc04578b



**DOI:** 10.1039/c6sc04578b

**Published:** 2016-12-15

**Authors:** Sebastian Bestgen, Olaf Fuhr, Ben Breitung, Venkata Sei Kiran Chakravadhanula, Gisela Guthausen, Frank Hennrich, Wen Yu, Manfred M. Kappes, Peter W. Roesky, Dieter Fenske

**Affiliations:** a Institute of Inorganic Chemistry , Karlsruhe Institute of Technology (KIT) , Engesserstraße 15 , 76131 Karlsruhe , Germany . Email: sebastian.bestgen@kit.edu ; Email: dieter.fenske@kit.edu; b Institute of Nanotechnology and Karlsruhe Nano Micro Facility (KNMF) , Karlsruhe Institute of Technology (KIT) , Hermann-von-Helmholtz-Platz 1, 76344 Eggenstein-Leopoldshafen , 76021 Karlsruhe , Germany; c Institute of Physical Chemistry , Karlsruhe Institute of Technology , Kaiserstraße 12 , 76131 Karlsruhe , Germany; d Helmholtz-Institute Ulm for Electrochemical Energy Storage (HIU) , Karlsruhe Institute of Technology (KIT) , 89081 Ulm , Germany; e Institute for Water Chemistry and Water Technology , Institute for Mechanical Process Engineering and Mechanics , Karlsruhe Institute of Technology , Adenauerring 20b , 76131 Karlsruhe , Germany; f Lehn-Institute for Functional Materials , School of Chemistry and Chemical Engineering , Sun Yat-Sen University , Guangzhou , People's Republic of China; g Zhejiang Provincial Key Laboratory for Chemical and Biochemical Processing Technology of Farm Products , School of Biological and Chemical Engineering , Zhejiang University of Science and Technology , No. 318 Liuhe Road , Hangzhou , 310023 , People's Republic of China

## Abstract

With the aim to synthesize soluble cluster molecules, the silver salt of (4-(*tert*-butyl)phenyl)methanethiol [AgSCH_2_C_6_H_4_^*t*^Bu] was applied as a suitable precursor for the formation of a nanoscale silver sulfide cluster. In the presence of 1,6-(diphenylphosphino)hexane (dpph), the 115 nuclear silver cluster [Ag_115_S_34_(SCH_2_C_6_H_4_^*t*^Bu)_47_(dpph)_6_] was obtained. The molecular structure of this compound was elucidated by single crystal X-ray analysis and fully characterized by spectroscopic techniques. In contrast to most of the previously published cluster compounds with more than a hundred heavy atoms, this nanoscale inorganic molecule is soluble in organic solvents, which allowed a comprehensive investigation in solution by UV-Vis spectroscopy and one- and two-dimensional NMR spectroscopy including ^31^P/^109^Ag-HSQC and DOSY experiments. These are the first heteronuclear NMR investigations on coinage metal chalcogenides. They give some first insight into the behavior of nanoscale silver sulfide clusters in solution. Additionally, molecular weight determinations were performed by 2D analytical ultracentrifugation and HR-TEM investigations confirm the presence of size-homogeneous nanoparticles present in solution.

## Abstract 2

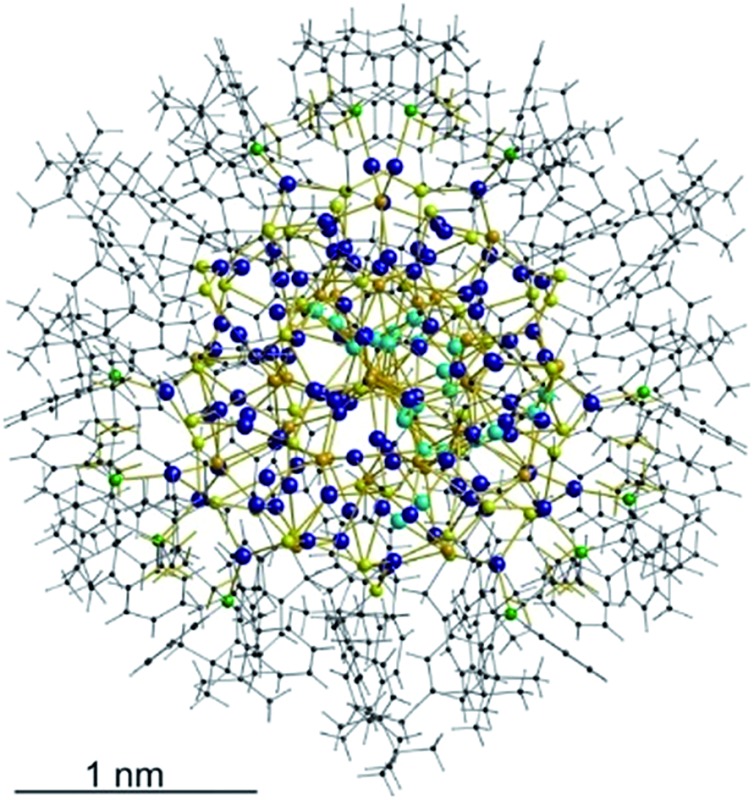
The soluble 115 nuclear silver cluster [Ag_115_S_34_(SCH_2_C_6_H_4_^*t*^Bu)_47_(dpph)_6_] was synthesized and fully characterized in solution and in the solid state.

During the last decades, synthetic pathways, structures and physical properties of nanoscale coinage metal chalcogenide clusters have been intensely investigated by many research groups.^1,2^ These molecules were thoroughly investigated in terms of their photophysical properties (*e.g.* photoluminescence) and act as molecular model systems for the study of quantum dots and quantum confinement effects.^3–6^ However, besides finding a suitable synthetic approach for clusters with more than a hundred heavy atoms, the characterization of these materials is very challenging and often exhausts the limits of physical methods like mass spectrometry. In the ideal case, single crystals suitable for X-ray crystallography are obtained which allows a full structural elucidation. Still, such X-ray analysis cannot be considered as trivial since spherical molecules are often disordered, modularized or twinned. Investigations of heavy organometallic or cluster-type molecules by mass spectrometry often fail for several reasons. Besides *e.g.* insolubility in common solvents, these compounds are often neutral species, which precludes simple electrospray transfer into the gas phase without decomposition. Although significant improvements in the field of high-resolution transmission electron microscopy (HR-TEM) have been achieved, the characterization of chalcogenide clusters with this method remains challenging, as can be seen below.^7^ Due to their low solubility, coinage metal chalcogenides were rarely characterized in solution. Only a very few ^1^H NMR but no heteronuclear measurements were reported.^8^ In general, these studies give only little insight into the properties of coinage metal chalcogenide clusters in solution.

Herein, we report on a nanoscale silver sulfide cluster, which was characterized in the solid-state by single crystal X-ray diffraction and in solution by heteronuclear NMR and further spectroscopic methods. Larger, hardly soluble nanoparticles like [Ag_320_(S^*t*^Bu)_60_S_130_(dpph)_12_] or [Ag_490_S_188_(S^*t*^C_5_H_11_)_114_] have been reported earlier by our group. They were obtained by the reaction of a suitable silver chalcogenide and S(SiMe_3_)_2_ in the presence of a mono- or bidentate phosphine.^9^ Their molecular structures are described as core–shell clusters, in which an inorganic core [Ag_2_S]_*x*_ is surrounded by silver thiolates and neutral phosphine ligands. Furthermore, the functionalization of the ligand shell with *e.g.* ferrocene units or amines was achieved.^10–12^ The kinetic stabilization of the silver chalcogenide core with space-filling ligands is of fundamental importance since small Ag_*x*_S_*y*_-particles are thermodynamically unstable.^13^ In addition, the applied phosphines and the organic substituent of the thiolate are responsible for the solubility properties of the cluster molecules. Silver thiolates are hardly soluble in common organic solvents. By the addition of phosphines to silver thiolates, small oligomeric clusters are formed, which are soluble in *e.g.* toluene.^14^ These species then react with the silylated chalcogenide source S(SiMe_3_)_2_ to form larger cluster species. It is assumed that there is a complicated equilibrium of different molecular species present in the initial reaction mixture, which finally leads to the crystallization of the most stable or least soluble product.^1^ Although clusters with bulky and solubility-enhancing groups like ^*t*^Bu or Np (neopentyl) were synthesized, they are generally poorly soluble and do not allow for further investigations in solution.^9^ However, in the present study we were able to isolate a novel silver sulfide cluster with enhanced solubility properties.

In order to increase the solubility of silver sulfide clusters, we chose (4-(*tert*-butyl)phenyl)methanethiol as building block for the synthesis of the silver compound [AgSCH_2_(C_6_H_4_)^*t*^Bu]. The thiolate was obtained as a colorless and air stable solid in very good yields.^15^ Subsequently, the silver thiolate was reacted with S(SiMe_3_)_2_ in the presence of 1,6-(diphenylphosphino)hexane (dpph) in toluene (Scheme 1).

**Scheme 1:**
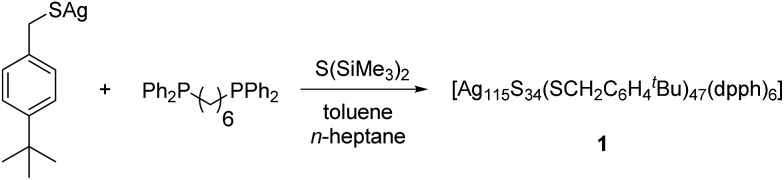
Synthesis of [Ag_115_S_34_(SCH_2_C_6_H_4_^*t*^Bu)_47_(dpph)_6_] **1**.

Within 7 days, the initial colorless solution turned to yellow, red and finally dark green. Then, the reaction mixture was dried under vacuum and extracted with *n*-heptane. Within a few days, the formation of dark green single crystals was observed (Fig. S1‡).

The structure and composition of the product was deduced by single crystal X-ray analysis. Compound **1** crystallizes in the triclinic space group *P*1 with one molecule in the asymmetric unit. The nanoscale molecule consists of 115 silver(i) ions, which are connected by 34 S^2–^ ions inside the core and coordinated by 47 thiolate ligands as well as 6 bidentate dpph ligands on the outer shell (Fig. 1). The composition of **1** was further confirmed by elemental analysis. In order to investigate the thermal stability of the compound, thermal gravimetric analysis (TGA) was conducted under a nitrogen stream and a heating rate of 2 K min^–1^ (Fig. S12‡). Following those results, the onset of decomposition occurs at approximately 160 °C and continues until 600 °C with a total weight loss of 47 wt%. This corresponds to a complete loss of the organic ligand shell as well as sulfur leaving elemental silver. The final residue is a ductile and metallic-shiny material with amorphous structure (following powder X-ray diffraction experiments). According to the crystallographically determined sum formula, the cluster itself is a neutral species. The diameter of the inorganic core ranges between 1.7 nm and 2 nm, but taking into account the outer ligand shell, a diameter between 3 nm and 3.3 nm is assigned. The sulfide ions coordinate to silver ions in bridging μ_2_, μ_3_, μ_4_, μ_5_, μ_6_, and μ_7_ modes, which leads to a broad range of Ag–S bond lengths. In the case of μ_2_-S, Ag–S bond lengths range from 2.34 Å to 2.58 Å (av. 2.41 Å).

**Fig. 1:**
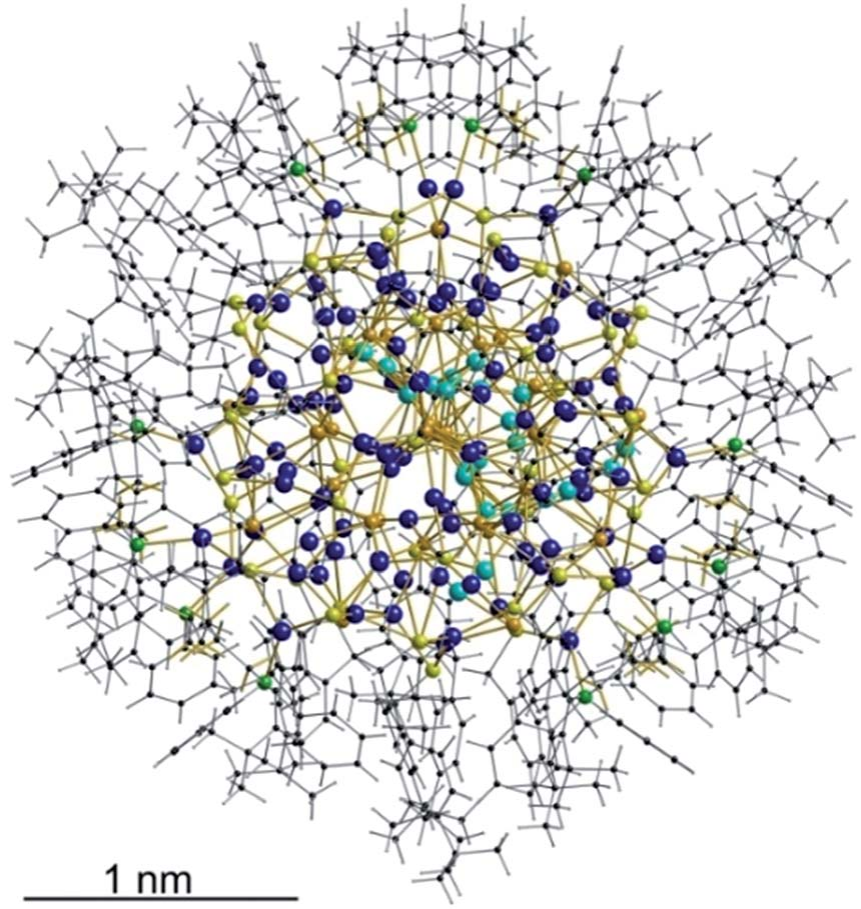
Crystal structure of [Ag_115_S_34_(SCH_2_C_6_H_4_^*t*^Bu)_47_(dpph)_6_] **1**. Silver blue, silver (disordered) turquoise, sulfur (RS^–^) yellow, sulfur (S^2–^) (orange) phosphorous green, carbon black, hydrogen white.

As expected, the Ag–S bond lengths for μ_3_-S are elongated to a mean distance of 2.53 Å and for higher bridging modes, even longer distances of av. 2.57 Å are observed. The intermetallic Ag–Ag distances (av. 2.8 Å) are in the expected range of similar silver sulfide clusters.^16^ Following previous theoretical investigations, argentophillic interactions^17,18^ or covalent bonds between the d^10^-metal ions are not anticipated.^13^ The dpph ligands are attached to 12 silver ions with Ag–P bond lengths of mean 2.40 Å, which is in the usual range for silver phosphine complexes.^19^ The phosphine coordinated silver ions feature different coordination modes: six silver ions are coordinated in trigonal planar fashion by two thiolate ligands and one phosphine with an ideal angular sum of 360°. The other six silver ions exhibit a distorted trigonal coordination sphere with angles between 339° and 351° or a tetrahedral coordination mode by three thiolate ligands and one phosphine. The combination of the symmetrically arranged phosphine ligands and the sterically demanding thiolate ligands allows an almost perfect shielding of the silver sulfide core (Fig. 2). Compound **1** features a silver sulfide (Ag_2_S)_34_ core, which is encapsulated by 47 silver thiolates [AgSCH_2_(C_6_H_4_)^*t*^Bu] and 6 phosphine ligands dpph. Therefore, **1** can also be formulated as [Ag_2_S]_34_@[(AgSCH_2_(C_6_H_4_)^*t*^Bu)_47_(dpph)_6_]. Silver ions inside the cluster core are exclusively surrounded by S^2–^ ions showing distorted linear, trigonal or tetrahedral coordination geometries. By taking a closer look at the chalcogenide ions, deltahedral substructures of the sulfur atoms are seen. Within the range of non-bonding S–S-distances between approx. 3 Å and 4 Å, two twofold bicapped hexagonal antiprisms formed by the sulfide ions, which blend into each other, can be deduced (Fig. 3).

**Fig. 2:**
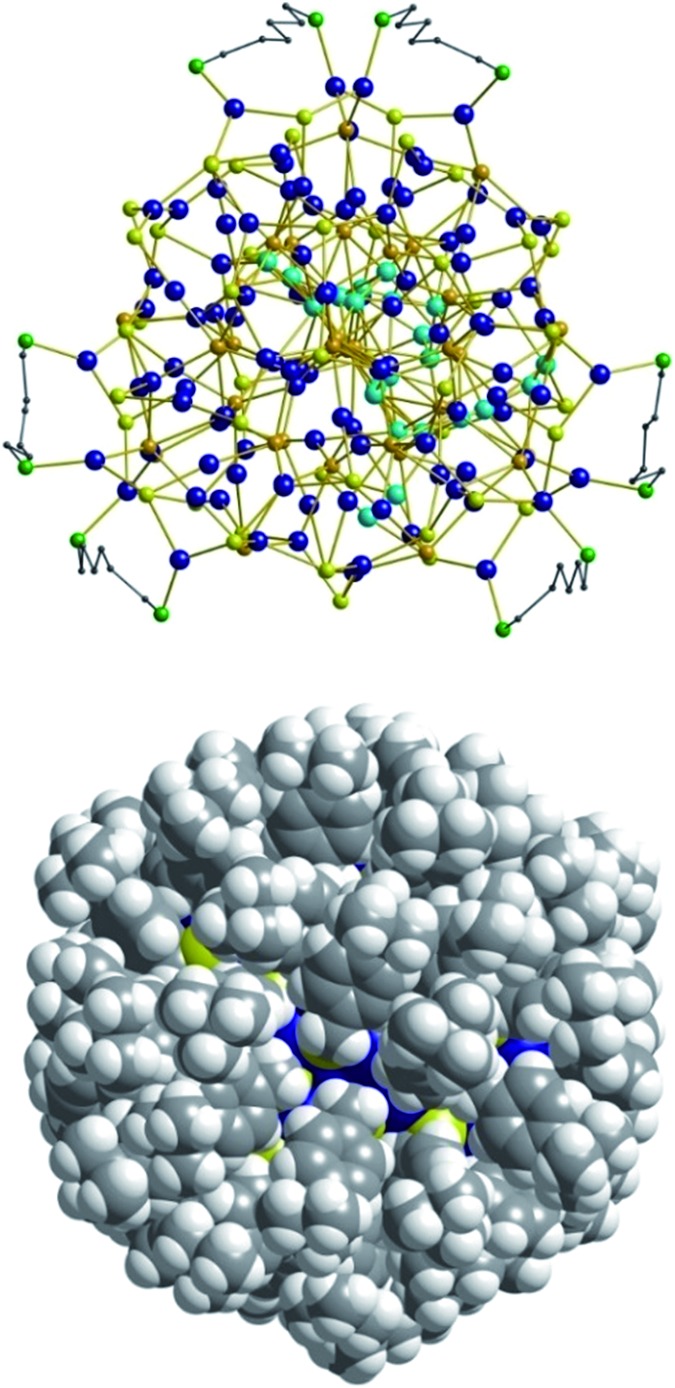
Top: Illustration of the inorganic cluster core of **1** with dpph ligands. Bottom: Space-filling model of the cluster surrounded by ^*t*^BuC_6_H_4_CH_2_S^–^ and dpph ligands.

**Fig. 3:**
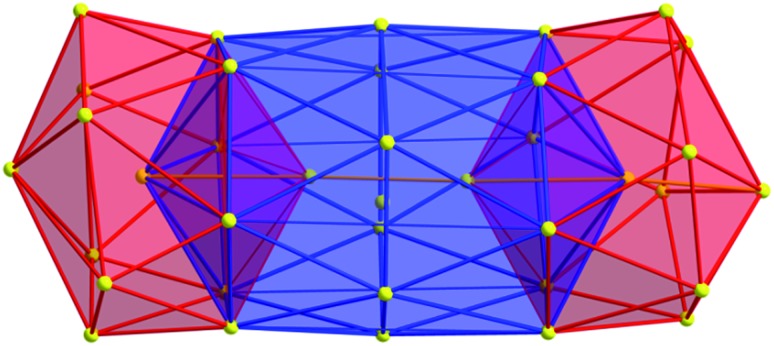
Cutout of the S-substructure within the Ag_115_ cluster. Sulfide ions form two twofold bicapped hexagonal antiprisms, which blend into each other (blue). Taking into account further sulphur atoms, the polyhedron is extended by two more identical, but distorted antiprisms. Depicted S–S bonds do not represent covalent bonds. Full S-substructure (Fig. S14‡).

In the center of each of the linearly fused antiprisms, a sulfide ion is located, which can be formulated as S@S_14_.^5^ Including the outer chalcogenide atoms, the polyhedron is extended by two more identical, but distorted capped hexagonal antiprisms. Hence, four S@S_14_ polyhedra interpenetrate each other in a linear fashion, which leads to a tubular S-substructure throughout the cluster. Polyhedra of this type are similar to frameworks, which are described as Frank–Kasper-polyhedra with the coordination number 14.^20,21^ These structures are found in *e.g.* Laves-phases.^1,22^ It is noteworthy that by looking at the extended solid state structure, the spherical molecules of **1** form a slightly distorted hexagonal close packing (hcp, Fig. S13‡).

Despite its size and in contrast to almost every other coinage metal chalcogenide cluster, **1** turned out to be very soluble in organic solvents like toluene, methylene chloride or chloroform. Based on this unexpected and unique solubility, further analysis of the cluster in solution was performed. Because of its intense color, UV-Vis spectra of **1** in CH_2_Cl_2_ were recorded (Fig. S2‡). A broad absorption in the range between 550 nm and 650 nm was observed, which corresponds to the absorption of red and yellow light and causes the dark green appearance of the compound both in the solid state and in solution. Following the UV-Vis spectra in the solid state (Fig. S3‡), the band gap of **1** amounts to 719 nm (1.72 eV) and is thus larger than that of bulk α-Ag_2_S (1 eV).^23^

The molecule was further investigated by NMR spectroscopic techniques. For comparison also ^1^H NMR spectra of [AgSCH_2_(C_6_H_4_)^*t*^Bu]_*x*_ and dpph were recorded (Fig. S5‡). The solutions of **1** in different solvents were monitored by NMR (one spectrum per hour) in order to determine their stability in solution, but no significant changes were observed for at least 24 hours (Fig. S6 and S7‡). However, after 3–4 weeks, a second set of resonances was observed in ^31^P{^1^H} NMR spectrum in chlorinated hydrocarbon solvents, which suggests rearrangement processes of the molecule. Unfortunately, no further characterization of this species was possible. NMR spectra were recorded both in CD_2_Cl_2_ and in CDCl_3_ and are fairly identical (Fig. S9 and S10‡). In the range between 0.75 ppm and 2.25 ppm, a few resonances were observed which were assigned to the *tert*-butyl groups of the thiolate ligand and C*H*_2_-moieties of the phosphine units. Between 3.3 ppm and 5.1 ppm, several multiplets are spread over 2 ppm and correspond to the SC*H*_2_-units of the thiolate, whereas the aromatic protons of both ligands were found in a range between 5.7 ppm and 8 ppm. By integration of the three areas in the ^1^H NMR, an integration ratio of 316 (C*H*): 94 (SC*H*_2_): 511 (C*H*_3_ + C*H*_2_) was found, which coincides well with the theoretically expected values for the sum formula deduced from XRD. Subsequently, **1** was also characterized by ^31^P{^1^H} NMR spectroscopy. Independent of the applied solvent, minor signals were observed in the ^31^P{^1^H} NMR spectra at 32 ppm and 42 ppm, which are most likely attributable to sulfonated phosphine species like dpphS_2_.^24^ A well resolved set of signals is observed in the region between –1 and +10 ppm (Fig. 4). Besides a signal at –16 ppm, which corresponds to the uncoordinated ligand dpph,^25^ the major signals have a hyperfine structure as result of a ^31^P and ^107/109^Ag coupling clearly showing the coordination of the dpph ligand to silver. Two doublets of doublets (dd) were detected at 0.9 ppm and 8.3 ppm. The coupling constants for each dd differ slightly and account for *J*(^31^P, ^109^Ag) = 540 Hz, 38 Hz (0.9 ppm) and *J*(^31^P, ^109^Ag) = 560 Hz, 40 Hz (8.3 ppm). Thereby, the coupling constants of 540 Hz and 560 Hz are in very good agreement with coupling constants observed in other silver–phosphine complexes, whereas the smaller coupling constants might be attributed to the coupling of ^31^P with a second silver or phosphorous atom over three bonds.^25–28^ The unequal chemical shifts imply that two chemically different P–Ag species are present in solution.

**Fig. 4:**
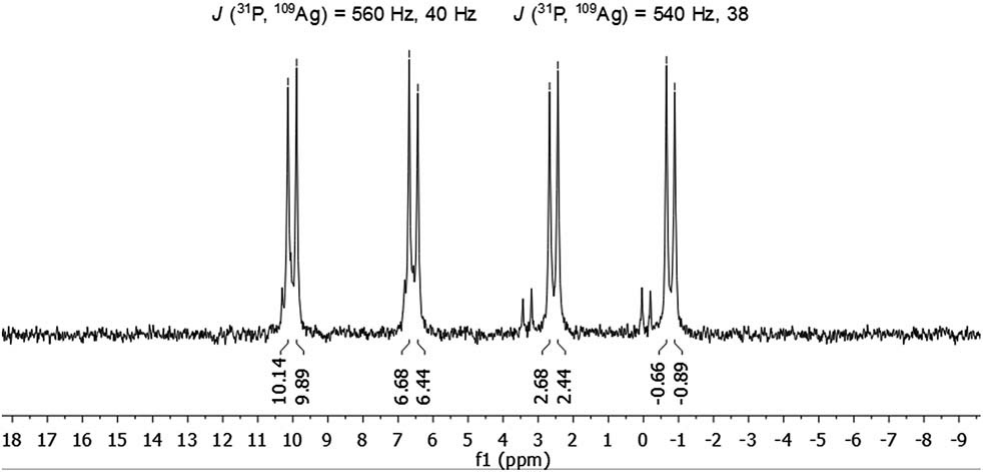
^31^P{^1^H} NMR spectrum of **1** in CDCl_3_.

This is probably explained by the already mentioned different coordination modes of the silver ions, which are directly attached to the phosphine ligands in a 1 : 1 ratio. It is noteworthy, that varying chemical shifts depending on the coordination behavior of the silver ions were also observed in polymeric silver–phosphine networks and in macrocyclic silver rings with long chain diphosphines.^19,25^

In addition to ^31^P{^1^H} NMR spectra, one- and two-dimensional ^109^Ag/^31^P NMR experiments were conducted (Fig. 5). Despite its slightly lower natural abundancy compared to ^107^Ag, ^109^Ag was monitored due to its higher receptivity. However, the relative receptivity of ^109^Ag is rather low and very long *T*_1_ relaxation times (*e.g.* 1115 s for AgNO_3_ in D_2_O) are observed, which hampers a straightforward and profound analysis of molecular silver compounds.^27,29,30^ In one-dimensional ^109^Ag NMR experiments, no unambiguously assignable resonance was observed between 0 ppm and +1500 ppm, although measurements were conducted for 14 days (Fig. S8‡). Besides the inherent problems with the silver nucleus itself, this could also be caused by fluxional behavior of the silver atoms in **1**, as *e.g.* silver ions are mobile in the bulk phase Ag_2_S.^31^ Hence, ^31^P/^109^Ag-HSQC experiments were conducted in order to increase the sensitivity with respect to silver ions. Interestingly, a correlation between the resonances in the ^31^P{^1^H} NMR and ^109^Ag was indeed detected giving further proof, that the spectroscopically monitored resonances correspond to a silver phosphine compound.

**Fig. 5:**
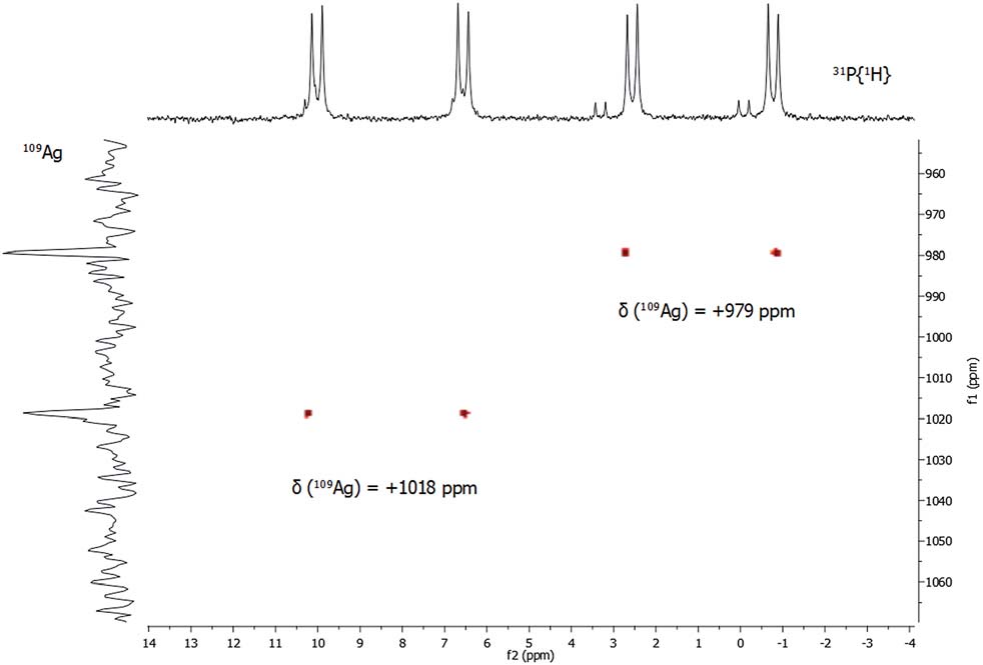
^31^P/^109^Ag gHSQC spectrum of **1** in CDCl_3_ at 297 K.

For each dd, crosspeaks were observed with ^109^Ag at chemical shifts of +979 ppm and +1018 ppm, respectively. This indicates again the presence of two chemically inequivalent silver–phosphine species but due to the small deviation in the chemical shifts, a very similar chemical environment is anticipated. According to ^109^Ag CP-MAS NMR spectra,^32^ the cross signals for **1** are located in the same region as those observed for solid silver thiolates, which suggests that soluble phosphine–silver thiolate species are observed in the NMR spectra of **1**. Additionally, diffusion-ordered NMR experiments (DOSY) were performed to determine the hydrodynamic radii of cluster molecules present in solution (see ESI‡). For all indexed peaks in the ^1^H NMR spectra both in the aromatic and in the aliphatic region (between 6 and 7.5 ppm as well as between 0.8 and 1.5 ppm), almost equal diffusion coefficients *D* of avg. 2.15 × 10^–10^ m^2^ s^–1^ were measured. Following the Stokes–Einstein equation, this diffusion coefficient of **1** corresponds to a hydrodynamic radius of 1.8 nm, which is in very good agreement with the crystallographically derived radius.

In order to further investigate whether discrete molecules of **1** (rather than aggregates or fragments thereof) are present in solution, the molecular mass of **1** was determined by analytical ultracentrifugation (Fig. S11‡).^33,34^ Fits of the measurements using the Lamm equation show that the solution comprises one dominant cluster species. This has a diffusion coefficient of 2.2 × 10^–10^ m^2^ s^–1^, which corresponds to a hydrodynamic diameter of 3.8 nm. This value is slightly larger compared to the crystallographically determined diameter of 3.3 nm but still in good agreement taking into account solvatization effects and the inaccuracy of the applied method. The diffusion coefficient and the hydrodynamic diameter are also very similar to the values obtained from the DOSY NMR investigations. The mass of this species is determined as 22 790 ± 500 Da, which agrees well with the exact mass of 24 688 Da found for **1** from X-ray crystallography. We attribute the difference to the potential loss of phosphine ligands based on our NMR investigations. However, a detailed analysis of the AUC data would include taking the compressibility of the toluene into account.^35^ This procedure, which is beyond the scope of the paper, would lead to slightly larger masses more in line with the crystallographically derived number.

To exclude a possible total decomposition of cluster **1** in organic solvents and to confirm whether nanoscale silver sulfide clusters are present in solution or not, high resolution TEM measurements (HR-TEM) were conducted. For this purpose, TEM samples were prepared by dissolving **1** in toluene and adding a drop of the solution (1.5 μL) onto a Cu 200 mesh TEM grid. The grid was washed three times with toluene (2 μL) afterwards. Since sulfur compounds are not stable when exposed to the electron beam,^36,37^ the expected particles in the micrographs should consist of Ag with trace amounts of S. Nevertheless the size of the particles provides information about the cluster core size. If a significant decomposition of the cluster in organic solvents occurs, the size of the remaining and/or reassembled particles should be significantly different from the values measured by XRD. The resulting HRTEM micrographs show crystalline Ag nanoparticles as expected. To further examine the elemental composition, large area energy dispersive X-ray spectroscopy (EDX) measurements were performed, which revealed the presence of Ag as well as S. Fig. 6 shows the TEM studies of the clusters originating from the toluene solution.

**Fig. 6:**
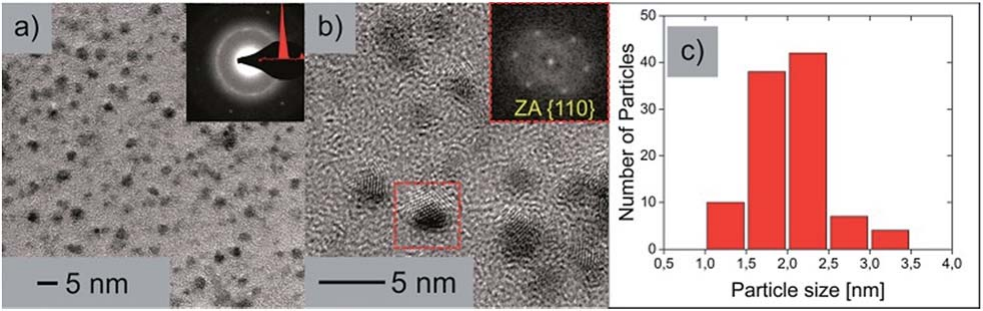
(a) Brightfield TEM image of the nanoparticles with an inset of the indexed SAED pattern. (b) HR-TEM micrograph of the nanoparticles clearly depicting the crystalline nature with an inset of the FFT from the marked area. (c) Particle size distribution.

In Fig. 6a an overview brightfield TEM image can be seen along with an inset of the selected area electron diffraction (SAED) pattern. From this indexed SAED pattern, *d*-values according to the increasing ring diameters from the center of the pattern of 2.37 nm and 1.46 nm correspond to the reflections of (111)_Ag_ and (022)_Ag_ respectively. Fig. 6b shows a HRTEM micrograph of the nanoparticles where the crystalline structure is clearly visible. An inset shows the corresponding fast Fourier transformation (FFT) of the marked nanoparticle in the zone axis of [100]. The particle size distribution (Fig. 6c) shows a vast majority of particles with sizes between 1.5 and 2.5 nm, which strengthens the results of the DOSY and AUC experiments, which claim that rather intact cluster molecules of **1** are present upon dissolution of the compound in organic solvents.

## Conclusions

In conclusion, we have prepared a new nanoscale silver sulfide cluster, which was characterized *via* multiple analytic techniques both in solution and in the solid state. The spherical molecule forms a hexagonal close packing throughout the crystal lattice with a diameter of 3.0–3.3 Å. The chalcogenide atoms inside the core are highly ordered and build up Frank–Kasper polyhedral-type cages with 14 vertices. The intensely green colored compound features a broad absorption in the UV-Vis range between 550 nm and 650 nm and is very soluble in *e.g.* CH_2_Cl_2_ and CHCl_3_. We think that this feature is caused by the thiolate ligand as it combines solubility-enhancing ^*t*^Bu-moieties with a flexible benzylthiol unit. This unique property of **1** allowed for an in-depth analysis *via* different NMR spectroscopic methods including ^31^P/^109^Ag gHSQC and DOSY NMR measurements. Based on those results, the cluster itself remains most likely intact. However, a certain amount of dissociation cannot be ruled out despite the results given above. By implication, this could also explain how such large molecules form: as many different fragments exist during the synthesis, they finally associate to form cluster molecules, which eventually crystallize. However, mass spectroscopic analysis *via* analytical ultracentrifugation proofed that intact nanoscale particles are present in solution. This result was backed up by DOSY NMR measurements, in which an identical diffusion coefficient was determined leading to a hydrodynamic radius, which corresponds well to the crystallographically determined one. Thus, both methods demonstrate a suitable method for the characterization of nanoscale particles and should be equally used in the analytics of such. Additionally, HR-TEM measurements were conducted, whereby the sample was taken from a cluster solution. Although the particle size distribution is in good agreement with the expectations based on single crystal X-ray analysis, the particles do not remain intact under the exposure of the electron beam. For instance, HR-TEM analysis revealed the formation of nanoscale crystalline silver particles indicating that decomposition and rearrangement processes took place under beam exposure. Hence, the analysis of nanoscale silver chalcogenides by TEM should always be combined by other analytic techniques. In summary, we think that an exact description of the behavior of macromolecular inorganic molecules in solution remains a challenging task but the combination of different analytic techniques allows for a deeper understanding of how these particles form and how they behave in solution. This understanding could be important for *e.g.* the application of nanoparticles in homo/heterogeneous catalysis or solubilized quantum dots.
