# Supplementary material for: [Ag115S34(SCH2C6H4 tBu)47(dpph)6]: synthesis, crystal structure and NMR investigations of a soluble silver chalcogenide nanocluster
Source: Chem Sci. 2016 Dec 15;8(3):2235–40. doi: 10.1039/c6sc04578b (PMC5408567; doi:10.1039/c6sc04578b)
Supplement: Supplementary file 1 [file SC-008-C6SC04578B-s001.pdf]

Electronic Supplementary Material (ESI) for Chemical Science.
This journal is © The Royal Society of Chemistry 2016

Supplementary Information for

$[Ag_{115}S_{34}(SCH_2C_6H_4{}^tBu)_{47}(dpph)_6]$: Synthesis, crystal structure and NMR investigations of a soluble silver chalcogenide nanocluster

Sebastian Bestgen,*[a] Olaf Fuhr,[bf] Ben Breitung,[b] Venkata Sei Kiran Chakravadhanula,[bd] Gisela Guthausen,[e] Frank Hennrich,[b] Wen Yu,[g] Manfred M. Kappes,[bc] Peter W. Roesky,[a] and Dieter Fenske*[abf]

[a] Dr. Sebastian Bestgen, Prof. Dr. Peter W. Roesky, Prof. Dr. Dieter Fenske
Institute of Inorganic Chemistry
Karlsruhe Institute of Technology (KIT)
Engesserstraße 15, 76131 Karlsruhe (Germany).
E-Mail: sebastian.bestgen@kit.edu
E-Mail: dieter.fenske@kit.edu

[b] Dr. Olaf Fuhr, Dr. Ben Breitung, Dr. Venkata Sai Kiran Chakravadhanula Dr. Frank Hennrich, Prof. Dr. Dieter Fenske
Institute of Nanotechnology (INT)
and
Karlsruhe Nano Micro Facility (KNMF)
Karlsruhe Institute of Technology (KIT)
Hermann-von-Helmholtz-Platz 1, 76344 Eggenstein-Leopoldshafen,
Mailbox 3640, 76021 Karlsruhe (Germany).

[c] Prof. Dr. Manfred M. Kappes
Institute of Physical Chemistry,
Karlsruhe Institute of Technology, Kaiserstr. 12,
76131 Karlsruhe, Germany.

[d] Dr. Venkata Sai Kiran Chakravadhanula
Helmholtz-Institute Ulm for Electrochemical Energy Storage (HIU), Karlsruhe Institute of Technology (KIT), 89081 Ulm, Germany.

[e] Prof. Dr. Gisela Guthausen
Institute for Water Chemistry and Water Technology and Institute for Mechanical Process Engineering and Mechanics, Karlsruhe Institute of Technology,
Adenauerring 20b, 76131 Karlsruhe, Germany

[f] Dr. Olaf Fuhr, Prof. Dr. Dieter Fenske
Lehn-Institute for Functional Materials, School of Chemistry and Chemical Engineering, Sun Yat-Sen University, Guangzhou, People's Republic of China.

[g] Dr. Wen Yu
Provincial Key Laboratory for Chemical and Biochemical Processing Technology of Farm Products, School of Biological and Chemical Engineering, Zhejiang University of Science and Technology, No. 318 Liuhe Road, Hangzhou, 310023, People's Republic of China.

Experimental Section

General procedures: All manipulations were performed under rigorous exclusion of moisture and oxygen in Schlenk-type glassware. Hydrocarbon solvents (toluene, *n*-pentane, *n*-heptane) were dried using an MBraun solvent purification system (SPS-800). Elemental analyses were carried out with an ElementarVario EL or Micro Cube. $[AgSCH_2C_6H_4{}^tBu]$ was synthesized following known procedures for silver thiolates from ${}^tBuC_6H_4CH_2SH$ and $AgNO_3$.[1] Bis(diphenylphosphino)hexane (dpph) was obtained from Sigma-Aldrich and used as received.

Synthesis of $[Ag_{115}S_{34}(SCH_2C_6H_4{}^tBu)_{47}(dpph)_6]$ (**1**):

The silver thiolate $[AgSCH_2C_6H_4{}^tBu]$ (0.35 g, 1.21 mmol, 1.00 eq.) and dpph (0.30 g, 0.66 mmol, 0.55 eq.) were suspended in toluene (30 mL). To the suspension, $S(SiMe_3)_2$ (0.09 mL, 76 mg, 0.43 mmol, 0.35 eq.) was added dropwise and a colour change from yellow to dark red was observed. The resulting clear solution was stirred for 7 days yielding a dark green solution. The solvent was removed under vacuum and the dark residue was extracted with *n*-heptane. After filtration, the formation of dark green needle-shaped crystals was observed on the wall of the Schlenk tube, which were separated from the mother liquor by decantation and finally dried under high vacuum. ^1H NMR ($CDCl_3$, 400 MHz): δ [ppm] = 0.50 – 1.85 (m, 511H, dpph-CH_2 and CH_3), 3.38 - 5.00 (m, 94H, thiolate-CH_2), 5.80 – 7.80 (m, 316H, CH). $^{31}P\{^1H\}$ NMR ($CDCl_3$, 160 MHz): δ [ppm] = 0.9 (dd, J = 540.0 Hz, J = 38.3 Hz), 8.3 (dd, J = 560.0 Hz, J = 39.9 Hz), $^{31}P/^{109}Ag$-HSQC-crosspeaks: ^{31}P dd @ 0.88 ppm and δ(^{109}Ag) = 979 ppm, ^{31}P dd @ 8.3 ppm and δ(^{109}Ag) = 1018 ppm. IR (ATR): $\tilde{\nu}$ (cm^{-1}) = 3050 (w), 3022 (w), 2952 (vs), 2903 (s), 2863 (s), 1511 (m), 1476 (m), 1461 (m), 1434 (m), 1410 (m), 1391 (m), 1361 (m), 1267 (m), 1228 (m), 1201 (m), 1104 (m), 1018 (m), 998 (w), 877 (w), 831 (m), 737 (m), 692 (s), 656 (m), 554 (m), 511 (m), 479 (w). UV−Vis (CH_2Cl_2): λ_{max} (nm) = 565 (broad). Elemental analysis calcd (%) for $[C_{697}H_{897}Ag_{115}P_{12}S_{81}]$ (24688.60): C 33.96, H 3.67, S 10.54; found C 34.02, H 3.442, S 9.91.

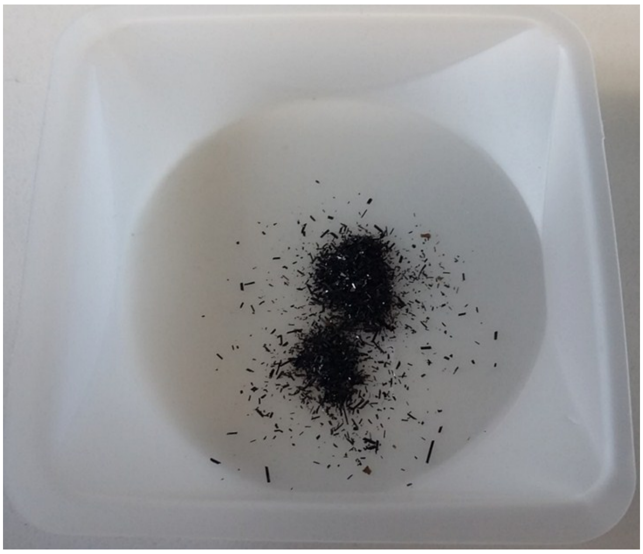

Figure S1: Representative picture of dried single crystals of **1** under air in a weighing boat.

UV-Vis measurements

UV-Vis spectra were recorded in CH_2Cl_2 using freshly prepared samples on a Varian Cary 50 spectrophotometer. UV-Vis measurements in the solid state were performed with a LAMBDA 900 spectrometer (Perkin-Elmer) from a suspension of grinded crystals in mineral oil between two quartz plates under air at room temperature. The absorption wavelength of the HOMO-LUMO gap was calculated by laying the tangent line through the inflection point of the first increase of the curve. The intersection point of the tangent with the abscissa was considered as wavelength of the HOMO-LUMO gap.

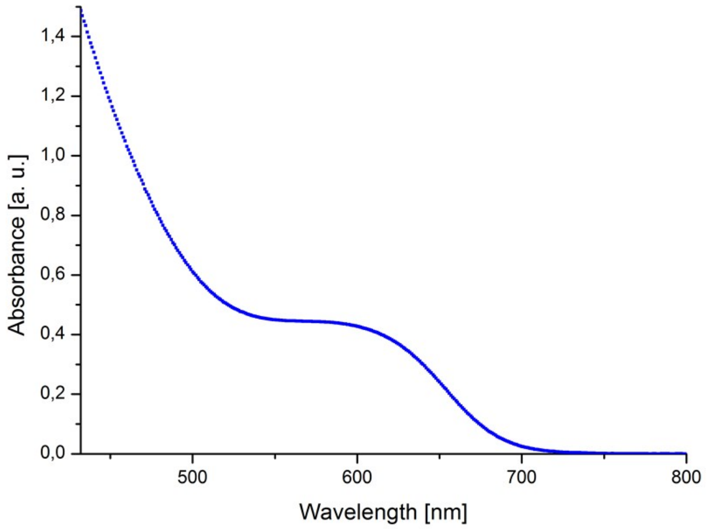

Figure S2: UV-Vis spectrum of **1** in CH_2Cl_2.

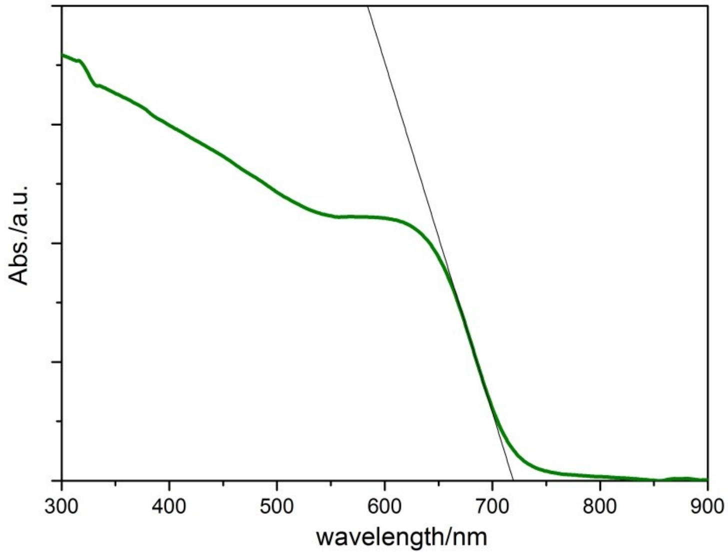

Figure S3: UV-Vis spectrum of **1** in the solid state.

IR-Spectra

IR spectra were obtained on a Bruker Tensor 37.

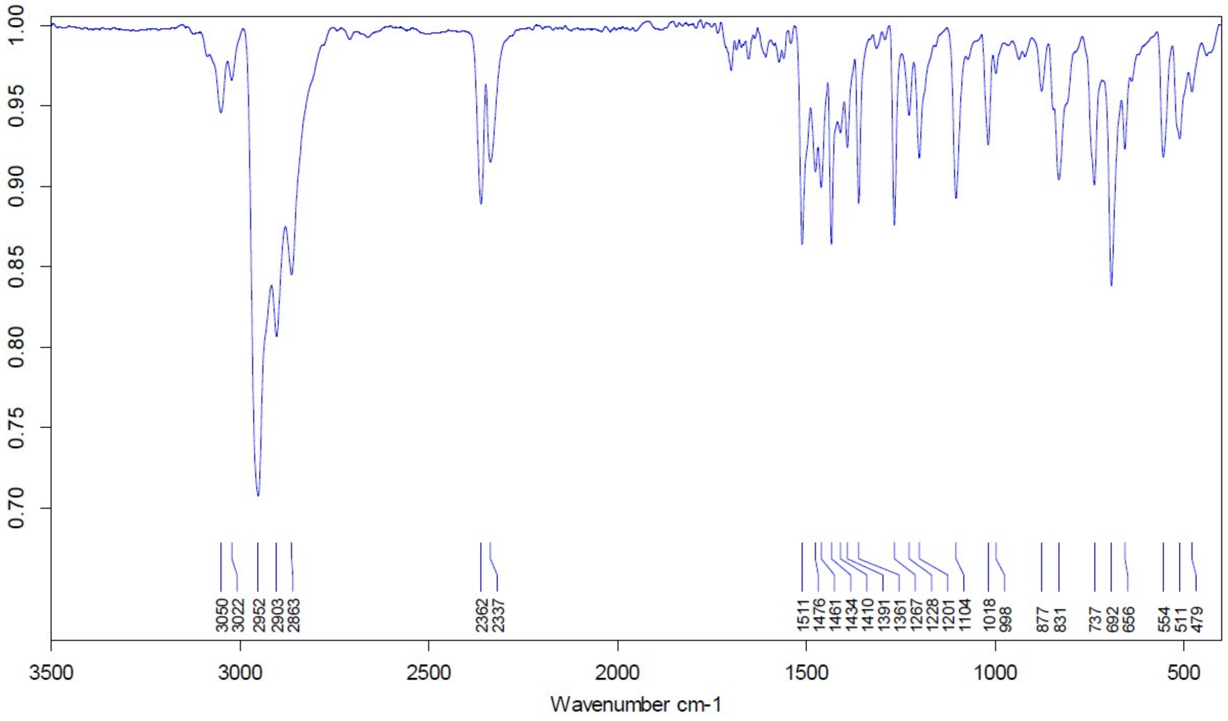

Figure S4: IR spectrum of cluster **1**.

NMR measurements

NMR spectra were recorded on a BrukerAvance II 300 MHz or Avance 400 MHz. Chemical shifts are referenced to the residual ^{1}H and ^{13}C resonances of the deuterated solvents and are reported relative to tetramethylsilane, relative to 85% phosphoric acid (^{31}P NMR) and to $AgNO_3$ in D_2O ($^{109}Ag\{^{1}H\}$ NMR).

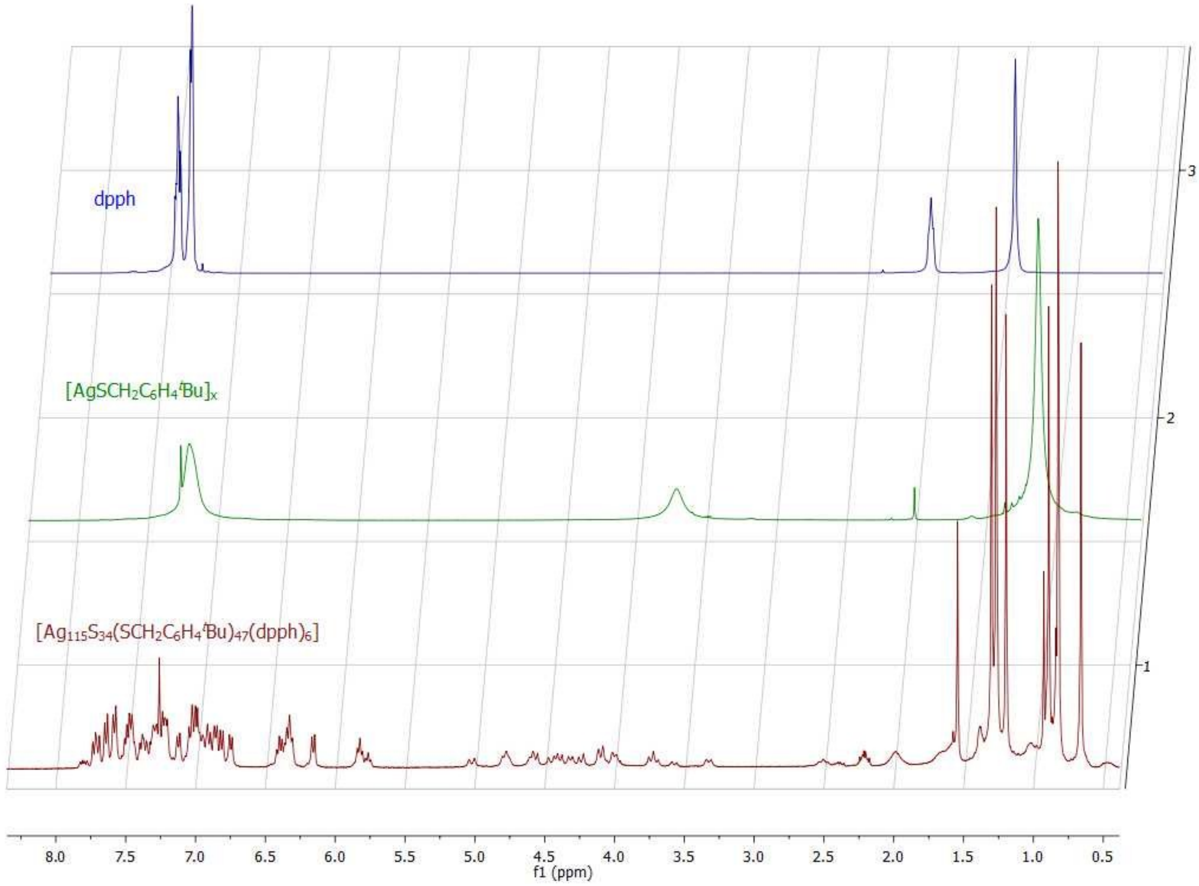

Figure S5: Stacked ^{1}H NMR spectra of bis(diphenylphosphino)hexane (blue), $[AgSCH_2C_6H_4{}^{t}Bu]$ (green), and **1** (red) in $CDCl_3$.

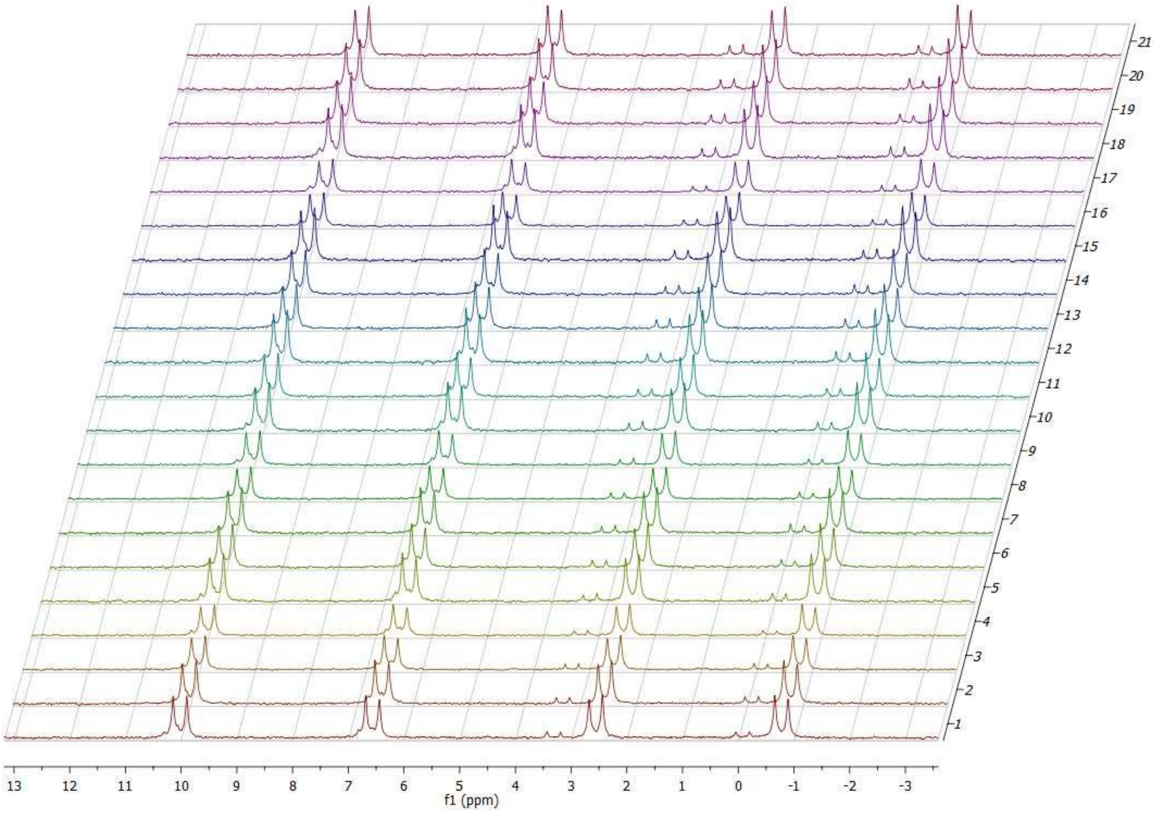

Figure S6: Stacked $^{31}P\{^{1}H\}$ NMR spectra of **1** measured every hour over a period of 21 hours in $CDCl_3$.

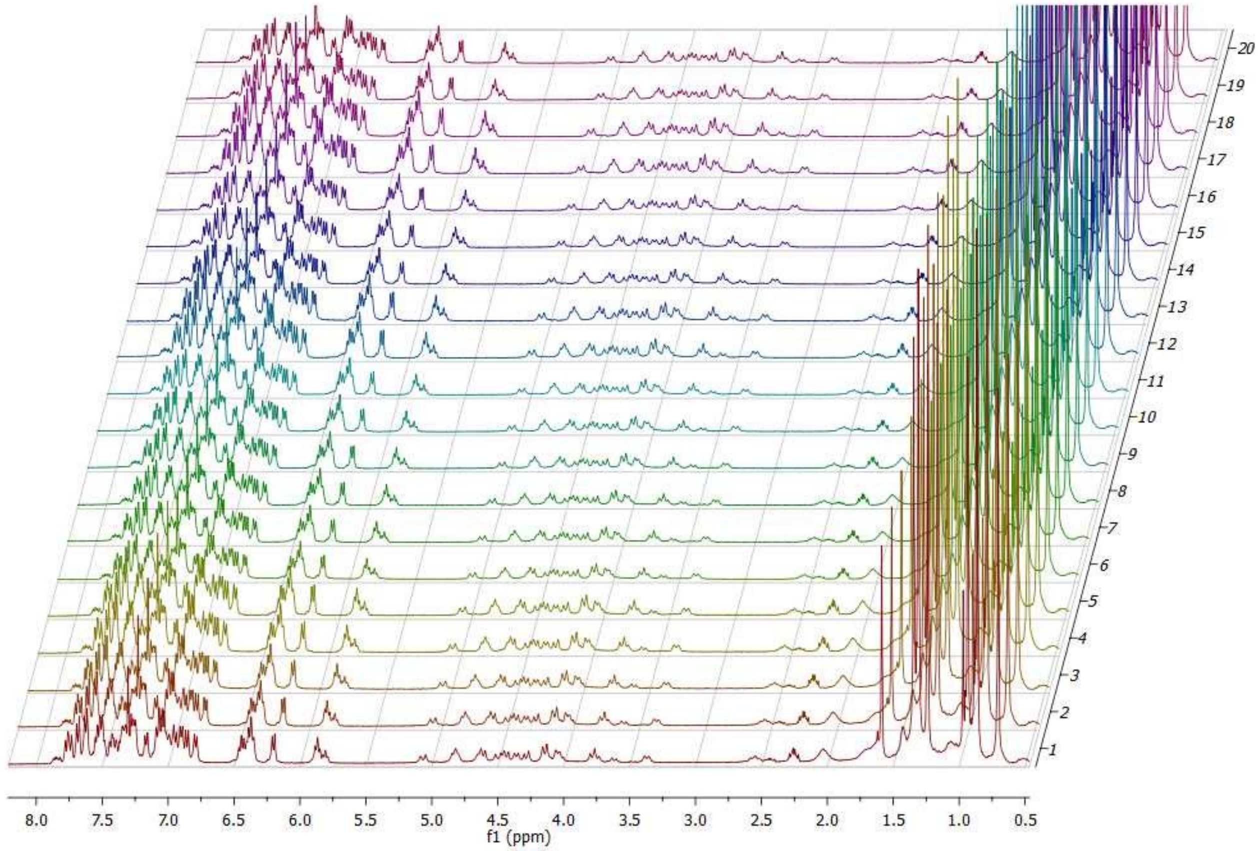

Figure S7: Stacked ^{1}H NMR spectra of **1** measured every hour over a period of 21 hours.

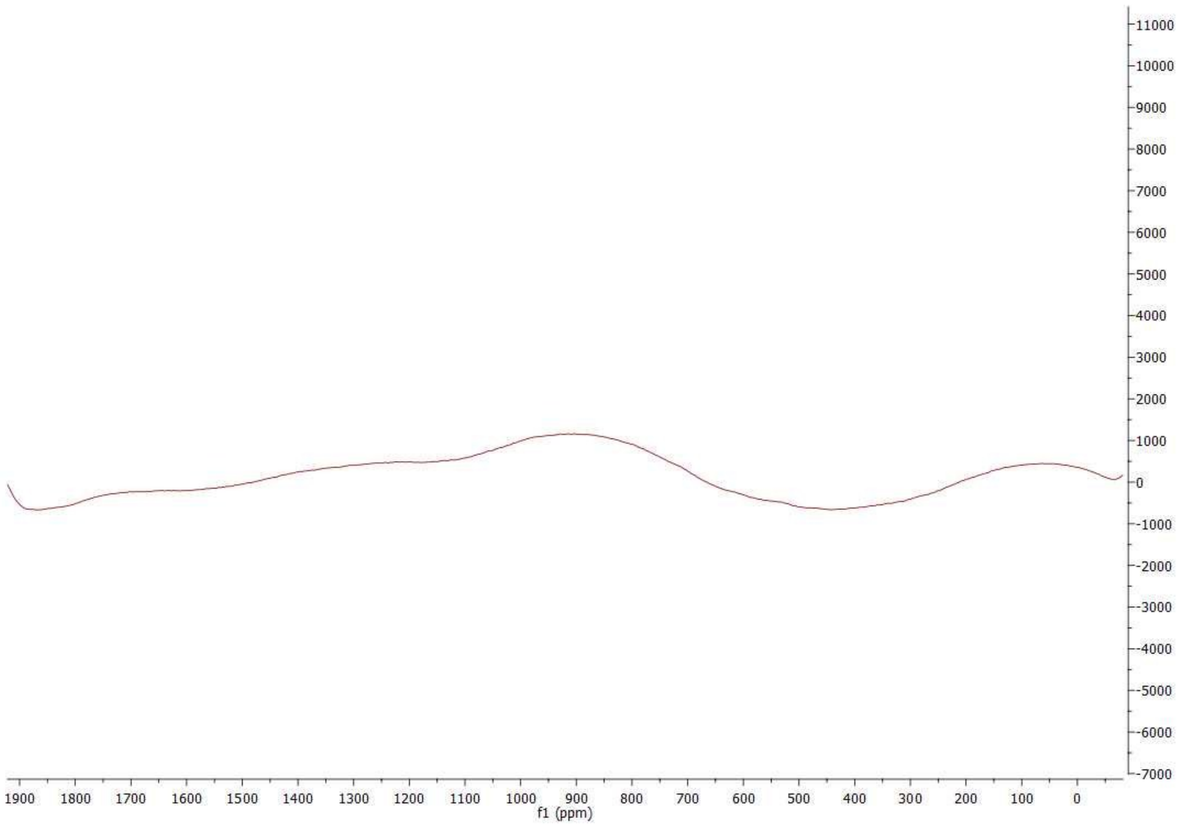

Figure S8: $^{109}Ag\{^{1}H\}$ NMR spectrum of **1** between -100 ppm ad +1900 ppm in $CDCl_3$.

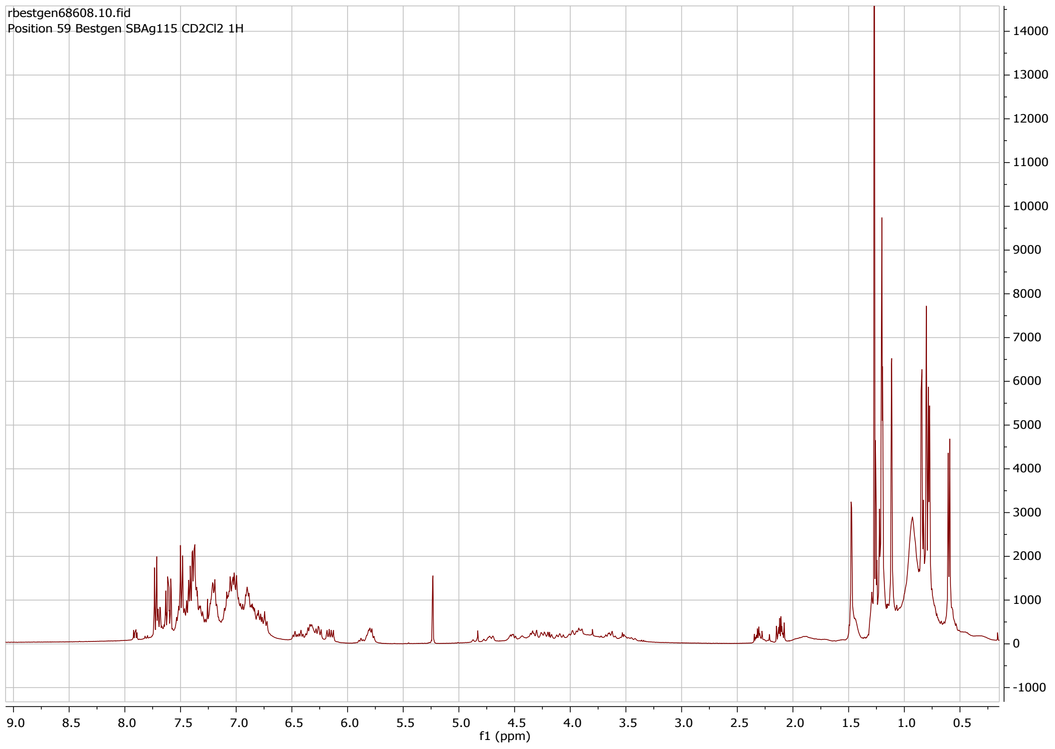

Figure S9: ^{1}H NMR spectrum of **1** in CD_2Cl_2.

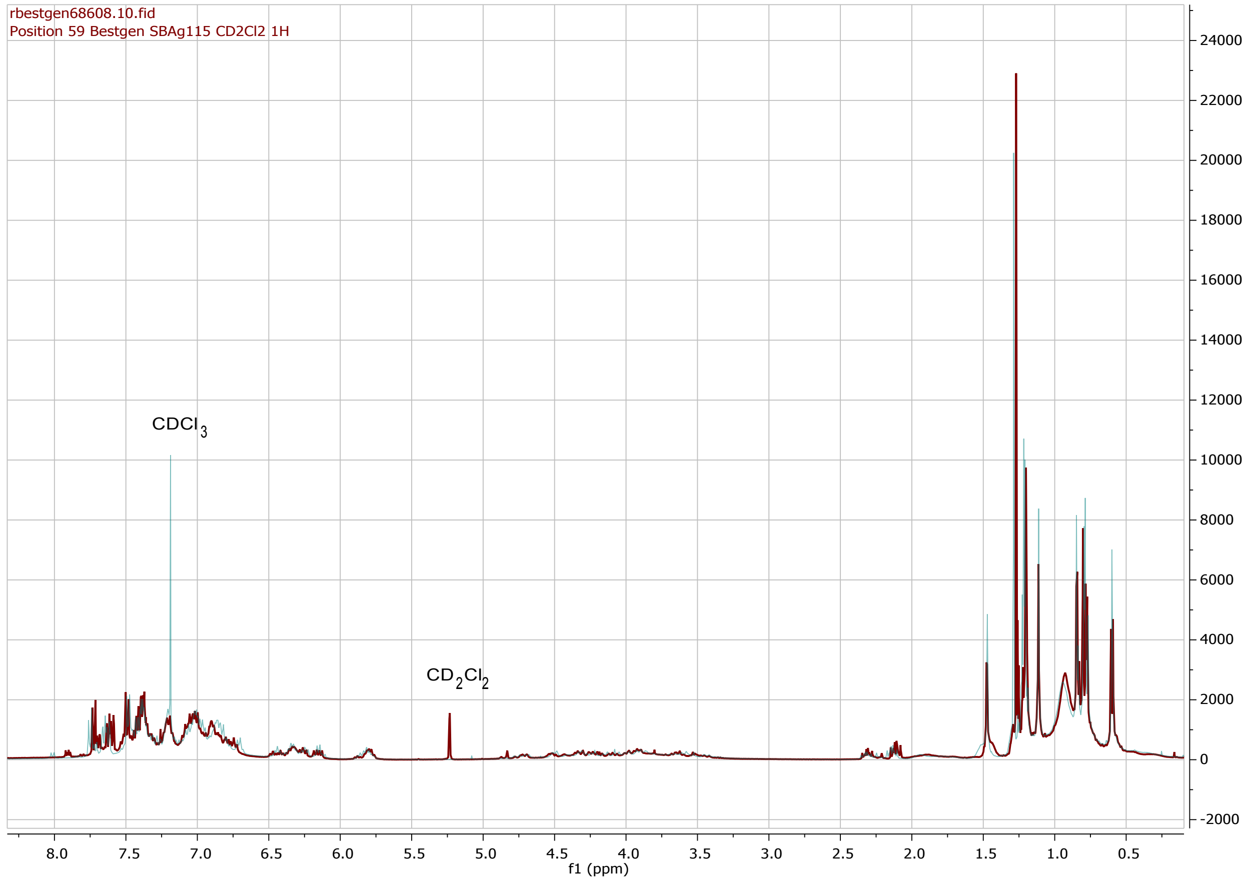

Figure S10: Superimposed ^{1}H NMR spectra of **1** in CD_2Cl_2 (red) and $CDCl_3$ (blue).

Diffusion-ordered NMR spectroscopy

^{1}H-NMR DOSY spectra were recorded on a Bruker Avance HD III WB 500 MHz spectrometer equipped with a broadband diffusion probe at Bruker, Rheinstetten. Pulsed field gradient echo was applied with a gradient duration of 2 ms, a diffusion time of 40 ms while varying the gradient amplitude. Both, ^{31}P and ^{1}H diffusion show same order of diffusion coefficients within the experimental error. Due to the better signal-to-noise ratio ^{1}H diffusion is shown here.

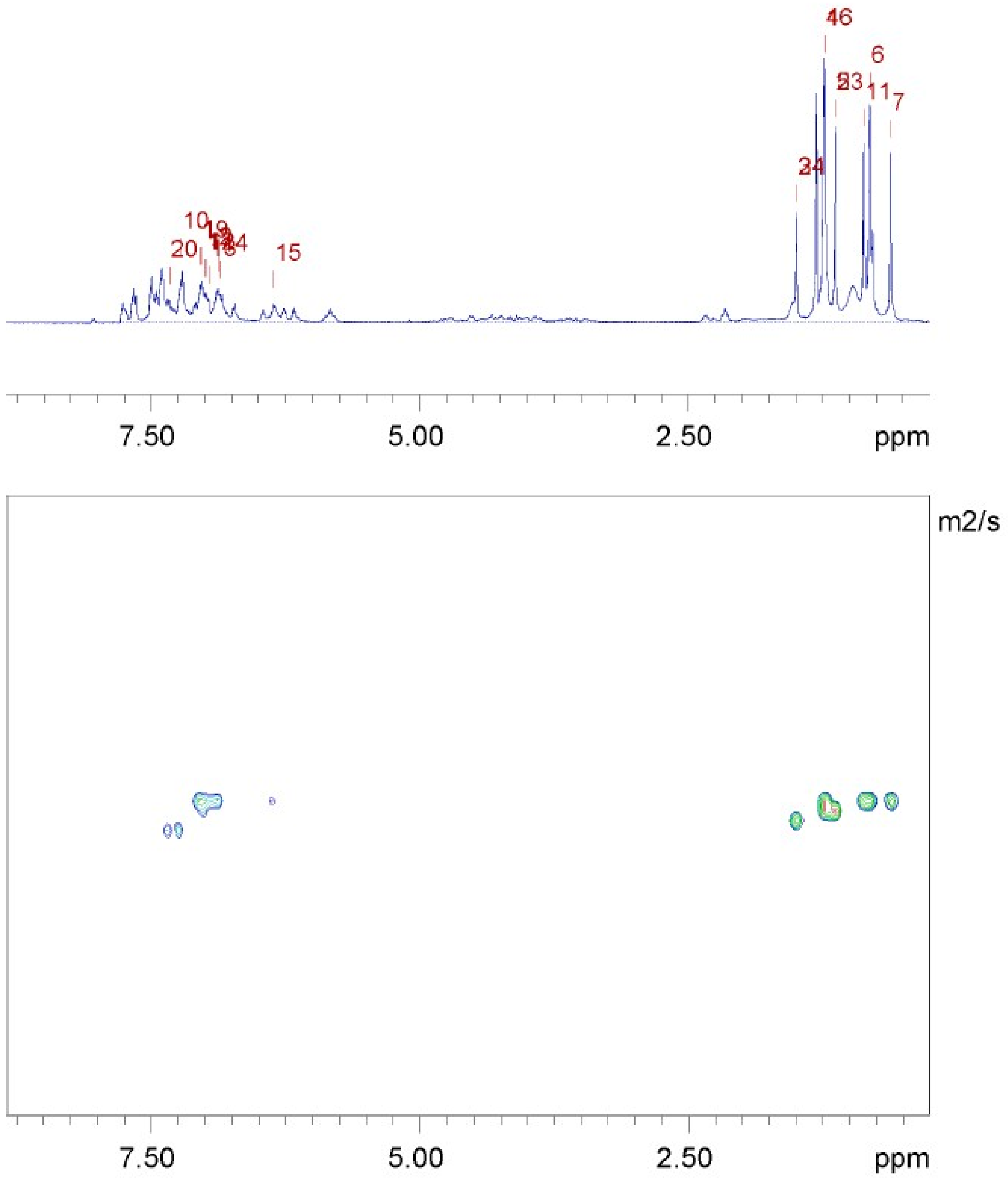

Fitted function:	f (x) = Io * exp (-D * x)
used gamma:	26752 rad/(s*Gauss)
used B values:	variable (gradient strength varied)
used little delta:	0.0020000 s
used big delta:	0.040000 s
Random error estimation of data:	RMS per spectrum (or trace/plane)
Systematic error estimation of data:	worst case per peak scenario
Fit parameter Error estimation method:	from fit using arbitray y uncertainties
Confidence level:	95%
Used peaks:	automatically picked peaks
Used integrals:	peak intensities
Used Gradient strength:	all values (including replicates) used

Peak name	F2 [ppm]	D [m2/s]	error
1	7.030	2.11e-10	1.658e-12
2	6.880	2.10e-10	1.773e-12
3	1.491	2.22e-10	1.506e-12
4	1.231	2.11e-10	1.303e-12
5	1.132	2.16e-10	1.164e-12
6	0.809	2.07e-10	9.615e-13
7	0.618	2.13e-10	1.138e-12
10	7.223	2.33e-10	4.588e-12
11	0.866	2.13e-10	1.100e-12
12	6.982	2.13e-10	2.396e-12
13	6.967	2.12e-10	3.115e-12
14	6.857	2.12e-10	2.018e-12
15	6.362	2.12e-10	2.439e-12
16	1.234	2.19e-10	1.452e-12
17	7.001	2.14e-10	2.763e-12
19	7.041	2.12e-10	1.715e-12
20	7.331	2.30e-10	5.532e-12
23	1.131	2.14e-10	1.650e-12
24	1.490	2.20e-10	1.620e-12

The viscosity η used in the Stokes–Einstein equation for $CDCl_3$ is 0.5559 x 10^{-3} $kgm^{-1}s^{-1}$. The viscosity was taken from www.knovel.com.

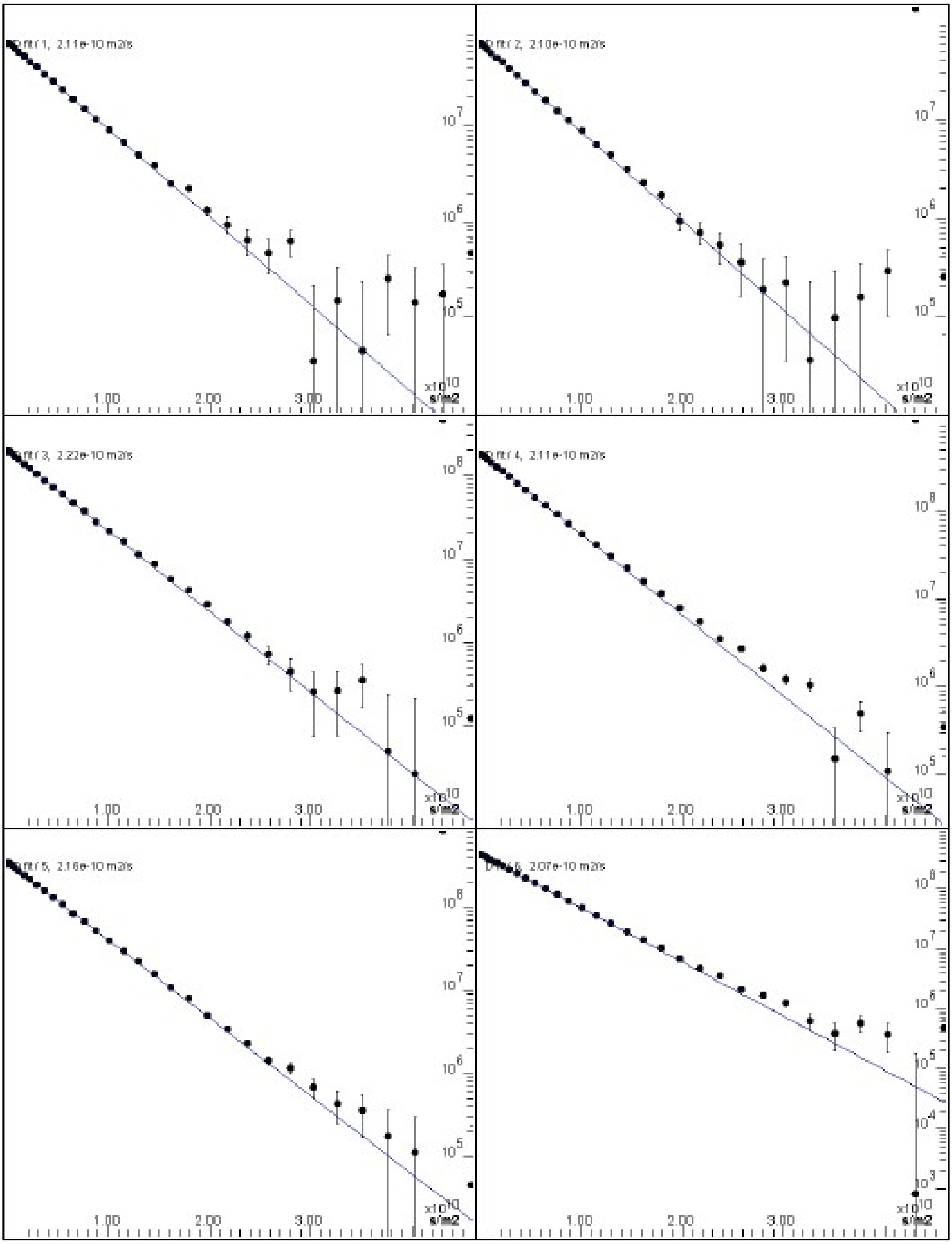
fit / 1, 2.11e-10 m2/s
fit / 2, 2.10e-10 m2/s
fit / 3, 2.22e-10 m2/s
fit / 4, 2.11e-10 m2/s
fit / 5, 2.16e-10 m2/s
D fit / 6, 2.07e-10 m2/s
1.00
2.00
3.00
s/m2

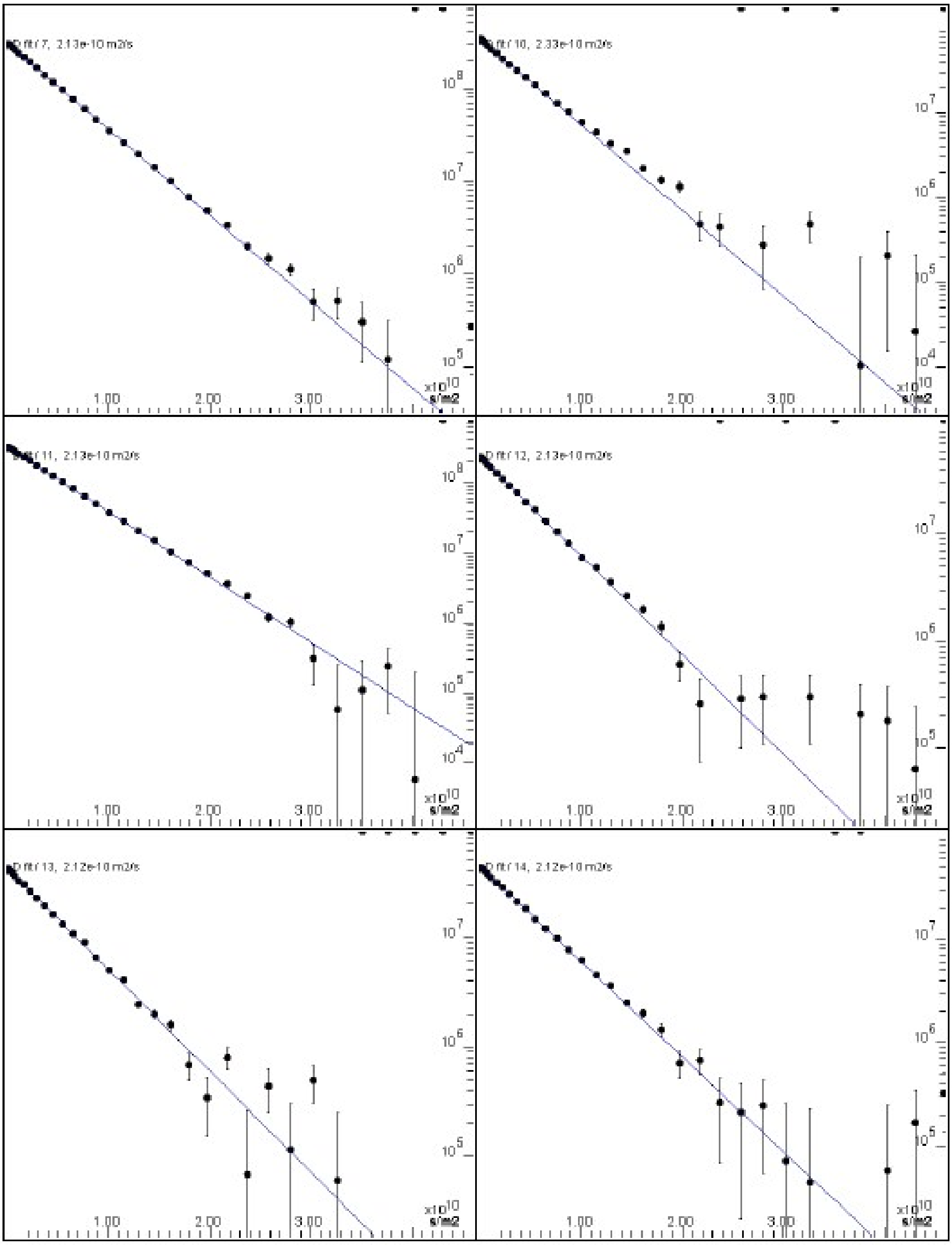
D fit / 7, 2.13e-10 m2/s
D fit / 10, 2.33e-10 m2/s
D fit / 11, 2.13e-10 m2/s
D fit / 12, 2.13e-10 m2/s
D fit / 13, 2.12e-10 m2/s
D fit / 14, 2.12e-10 m2/s
1.00
2.00
3.00
x10^10
s/m2

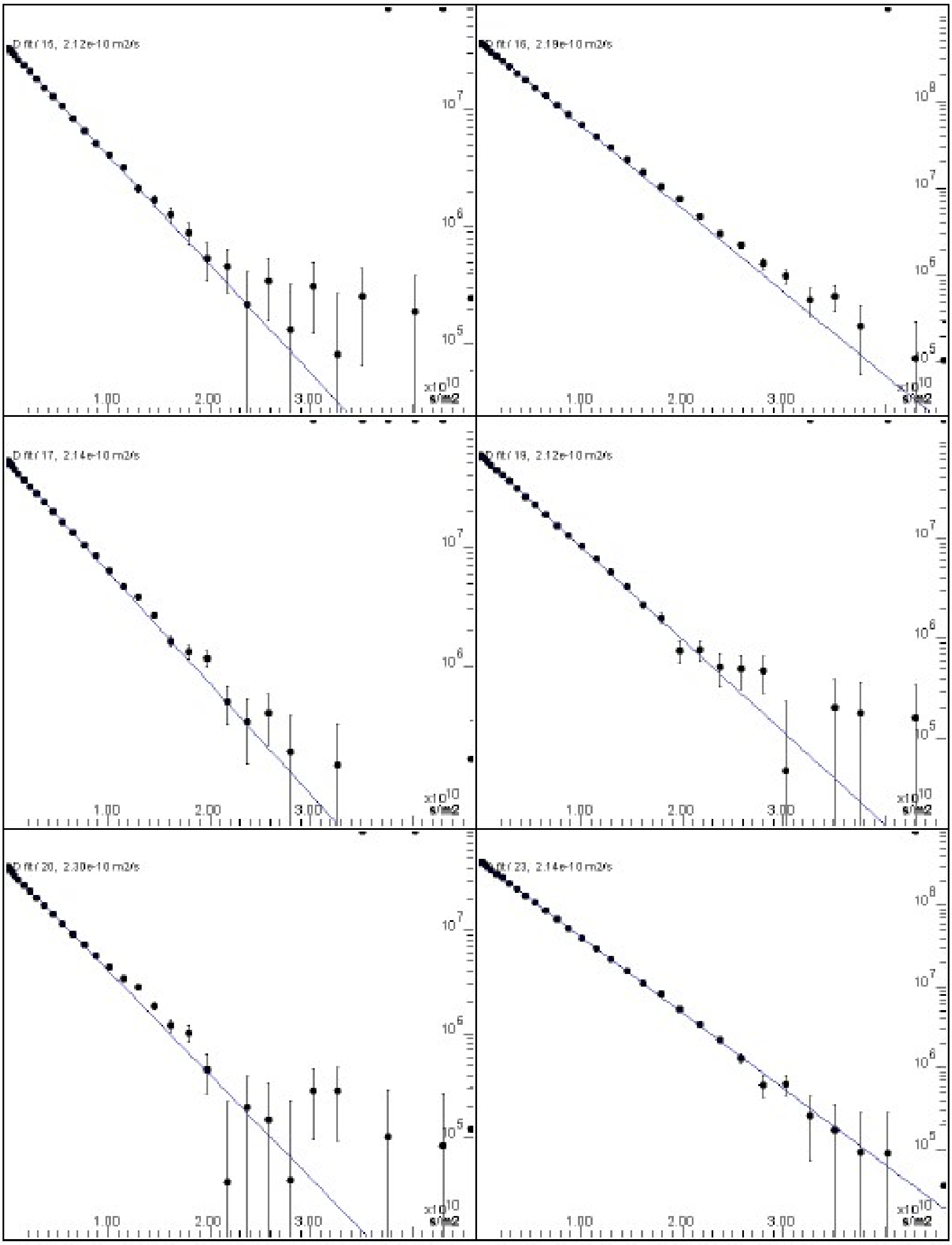
D fit / 16, 2.19e-10 m2/s
D fit / 17, 2.14e-10 m2/s
D fit / 19, 2.12e-10 m2/s
D fit / 20, 2.30e-10 m2/s
D fit / 23, 2.14e-10 m2/s
1.00
2.00
3.00
x10^10
s/m2

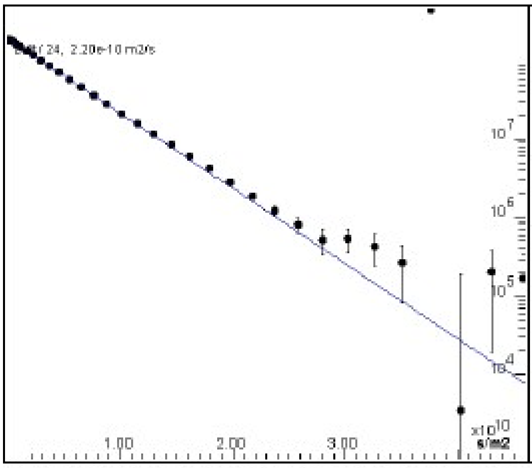
24, 2.20e-10 m2/s
10^7
10^6
10^5
10^4
1.00
2.00
3.00
x10^{10}
s/m2

Analytical ultracentrifugation (AUC)

For all AUC experiments we used a Beckman Coulter (Proteomelab XL-A/XL-I) analytical ultracentrifuge equipped with an AN-50 rotor and an optical detection system. Equal aliquots (440 ml) of $[Ag_{115}S_{34}(SCH_2C_6H_4{}^{t}Bu)_{47}(dpph)_6]$ in toluene and appropriate reference solvent were injected into a two-sector cell (12 mm optical path length) comprising sapphire windows and an Epon charcoal-filled centerpiece. Data were collected in sedimentation velocity mode with the absorption optics set to a detection wavelength of 320 nm where the sedimentation process is monitored by scanning the concentration profile, c(r, t), with respect to the radial distance from the rotor (r) and time (t). All experiments were performed at 40,000 rpm with a radial resolution of 0.001 cm; scans were recorded every 10 min for 4 hrs. The numerical fitting software SEDFIT was used to fit the absorbance profiles with Lamm's equation solutions to calculate the distribution of sedimentation and diffusion coefficients.[2, 3] A typical 2D SEDFIT screen capture is shown in Fig. S9. Size distribution and molar mass of $[Ag_{115}S_{34}(SCH_2C_6H_4{}^{t}Bu)_{47}(dpph)_6]$ cluster was then calculated according to the procedure described by Carney *et al.*

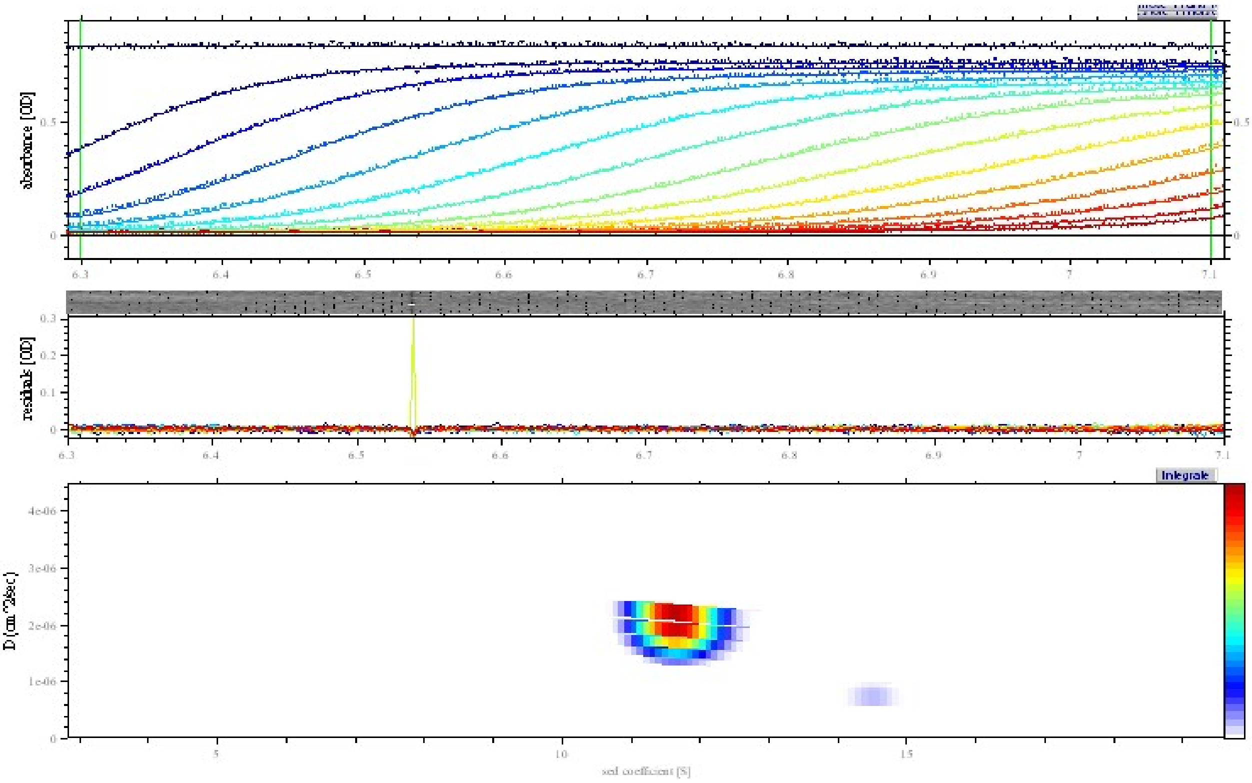

Figure S11: Screenshot 2-D sedimentation and diffusion coefficient distribution as fitted by SEDFIT.

We have analyzed our sedimentation velocity data to yield distributions of diffusion (D) and sedimentation (s) coefficients – using the method recently applied by Carney *et al.* to characterize dissolved gold nanoparticles.[4] This method numerically fits the experimental data using Lamm's equation and a two-dimensional (2D) model for s and D coefficient

distributions. The approach does not require a priori knowledge of particle density but instead offers an independent way of estimating it in addition to the particle molecular mass and its hydrodynamic radius. This is particularly useful for the characterization of core–shell nanoparticles because particle composition (i.e. shell thickness) can be estimated from these three numbers by mass conservation. There are specific assumptions inherent in this approach: (i) diffusion and sedimentation coefficient distributions are independent and (ii) diffusion contributions are fully resolved experimentally.

Figure S9 shows the resulting best fit to the AUC data of diffusion (D) and sedimentation (s) coefficient distributions. From these we have determined the average mass of the species present in solution. The mass distribution is dominated by a single peak centered at 22790 ± 500 Da. Hence, the particle has a diffusion coefficient of 2.2 x 10^{-10} m^2s^{-1}, which corresponds to a hydrodynamic diameter of 3.8 nm. Note: AUC using methylene chloride or chloroform was not possible because of centrifugation tube incompatibility

Electron microscopy

All the transmission electron microscopy measurements were performed using an aberration corrected FEI Titan 80-300 operated at 80 kV. Samples were prepared by dissolving them in toluene and adding one drop (1.5 µL) onto a Cu 200 mesh TEM grid. The grid was washed three times with toluene (2 µL), dried on air and inserted into the microscope.

Thermogravimetric analysis

Thermogravimetric analysis was performed using a Netzsch STA 409 C/CD device. The sample (20.900 mg) was heated under N_2 (25 l/min) from 300 K to 1000K with a heating rate of 2 K/min.

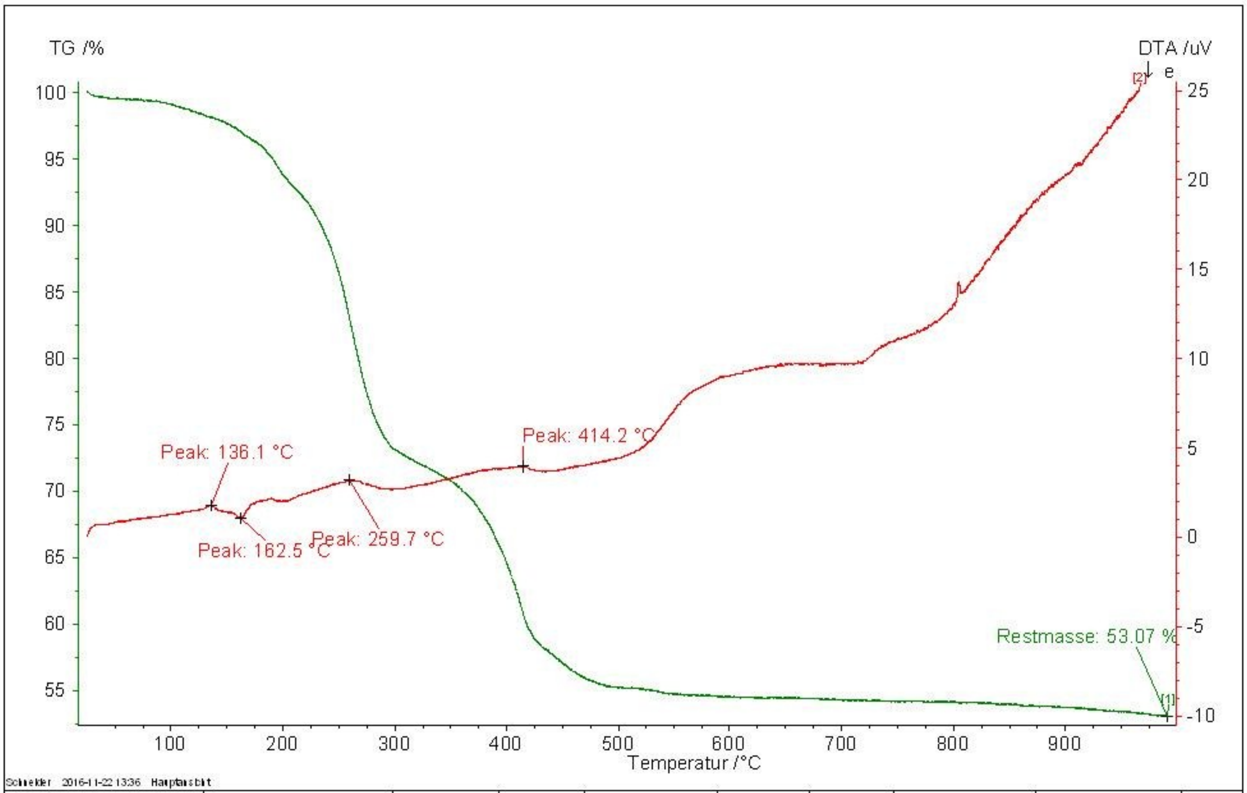

[#] Gerät	Datei	Versuchs-ID	Probe	Datum	Masse	Segment	Bereich	Atmosphäre	Korr.
[1] NETZSCH STA 409 C/CD	TGsubtrSBAg115_2... .mss	SBTGA	SBAg115	2016-11-17 15:27	20.900 mg	1/1	30/2.0(K/min)/1000	N2/25 / ---/--- /---/---	---
[2] NETZSCH STA 409 C/CD	DTAsubtrSBAg115_... .mss	SBTGA	SBAg115	2016-11-17 15:28	20.900 mg	1/1	30/2.0(K/min)/1000	N2/25 / ---/--- /---/---	---

Figure S12: TGA analysis of **1**. At 1000°C, the residual mass is 53.07% of the mass at the beginning of the measurement.

Crystallographic information

Low temperature single crystal X-ray diffraction was performed on a STOE STADIVARI diffractometer using Cu-Kα-radiation (λ = 1.54178). Using Olex2,[5] the structures were solved with the ShelXD[6] structure solution program the using Dual Space method and refined with the ShelXL[7] refinement package using Least Squares minimization. Hydrogen atoms were calculated on idealized positions. CCDC 1507868 contains detailed crystallographic data of this article, which can be obtained free of charge from the Cambridge Cristallographic Data Centre, www.ccdc.cam.ac.uk/data-request/cif.

Identification code	**1**
Empirical formula	$C_{697}H_{897}Ag_{115}P_{12}S_{81}$
Formula weight	24648.64
Temperature/K	150.15
Crystal system	triclinic
Space group	*P*1
a/Å	29.4745(4)
b/Å	35.8121(5)
c/Å	48.4263(7)
α/°	86.3790(10)
β/°	75.6370(10)
γ/°	74.1640(10)
Volume/Å^3	47638.4(12)
Z	2
ρ_{calc}g/cm^3	1.718
μ/mm^{-1}	20.594
F(000)	23920.0
Crystal size/mm^3	0.25 × 0.19 × 0.12
Radiation	CuKα (λ = 1.54178)
2Θ range for data collection/°	3.174 to 110.698
Index ranges	-14 ≤ h ≤ 31, -37 ≤ k ≤ 38, -51 ≤ l ≤ 51
Reflections collected	294599
Independent reflections	117059 [R_{int} = 0.1068, R_σ = 0.1127]
Independent reflections with I ≥ 2σ (I)	55411
Data/restraints/parameters	117059/326/4246
Goodness-of-fit on F^2	1.158
Final R indexes [I ≥ 2σ (I)]	R_1 = 0.1217, wR_2 = 0.3358
Final R indexes [all data]	R_1 = 0.1860, wR_2 = 0.3793
Largest diff. peak/hole / e Å^{-3}	5.63/-3.47
CCDC number	1507868

Though many crystals of **1** were X-rayed, refinement of the datasets was problematic due to significant disorder in the cluster core (especially the Ag positions) as well as in the ligand shell. The positions of all non-carbon were found in the differential Fourier map but

refinement was only possible with a significant number of constraints and restraints. Still there are high peaks of remaining electron density inside the cluster core indicating additional disorder of the silver atoms which was not further modelled. The contribution of the electron density of lattice bound toluene molecules in the voids between the cluster molecules was calculated using the SQUEEZE algorithm[8] and the hkl file was modified. GOF and R values in the table base on the modified data.

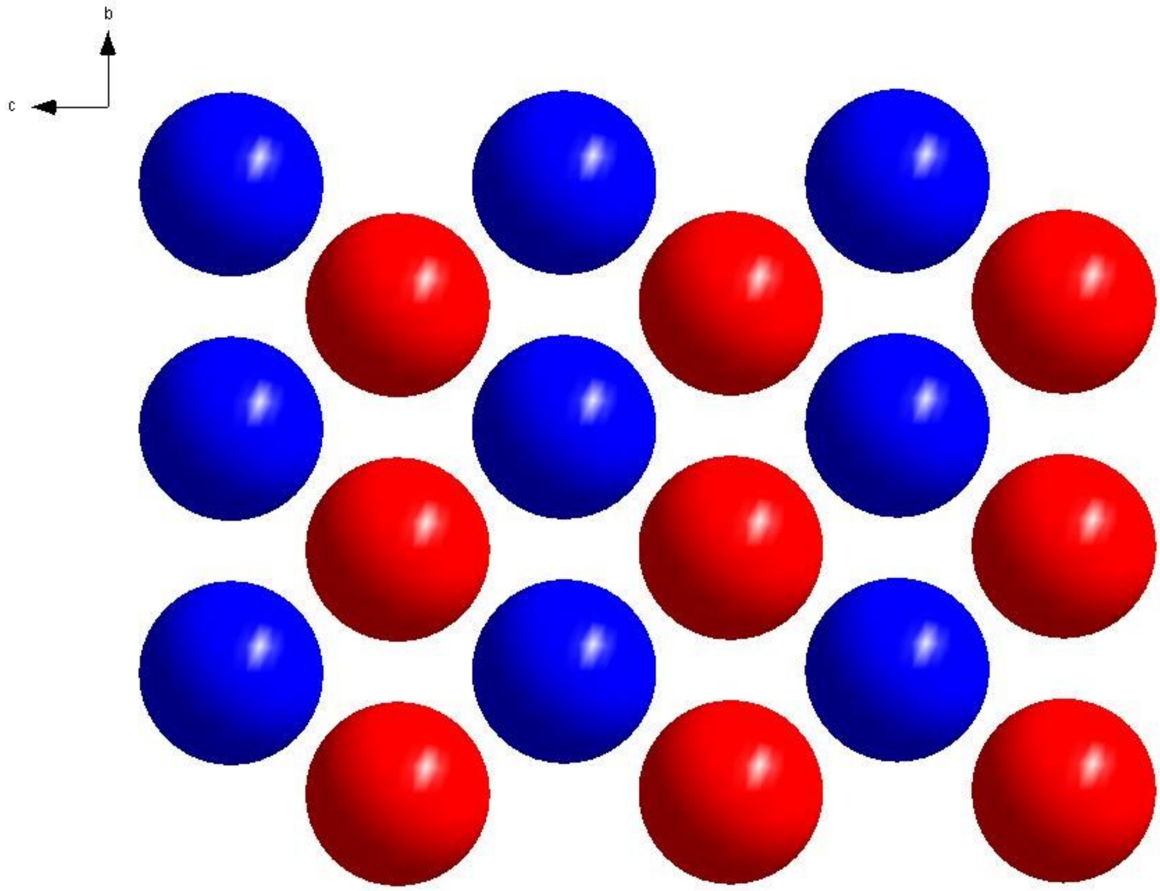

Figure S13: Extended solid state structure of **1**. The cluster molecules are illustrated as dummy atoms with r = 1.3 nm which were positioned in the center of the original cluster. Their arrangement corresponds to a distorted *hcp* with ABAB… layers highlighted in red and blue.

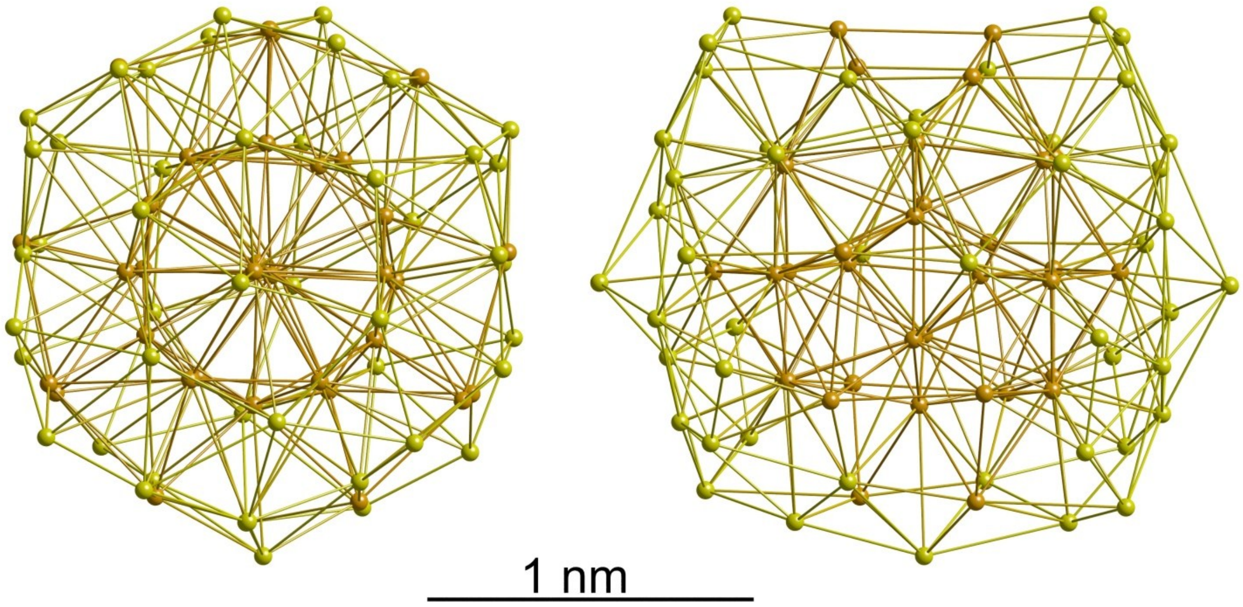

Figure S14: Complete S-substructure of **1**. S^{2-} (light brown), S in RS^- (yellow). Lines between S atoms are nonbinding and solely illustrate the geometric arrangement.

References

1. I. G. Dance, L. J. Fitzpatrick, A. D. Rae and M. L. Scudder, *Inorg. Chem.*, 1983, **22**, 3785-3788.
2. P. Schuck, *Biophys. J.*, **78**, 1606-1619.
3. J. Dam and P. Schuck, in *Methods Enzymol.*, Academic Press, 2004, vol. Volume 384, pp. 185-212.
4. R. P. Carney, J. Y. Kim, H. Qian, R. Jin, H. Mehenni, F. Stellacci and O. M. Bakr, *Nat. Commun*, 2011, **2**, 335.
5. O. V. Dolomanov, L. J. Bourhis, R. J. Gildea, J. A. K. Howard and H. Puschmann, *J. Appl. Crystallogr.*, 2009, **42**, 339-341.
6. G. Sheldrick, *Acta Crystallogr. Sect. A*, 2008, **64**, 112-122.
7. G. Sheldrick, *Acta Crystallogr. Sect. C*, 2015, **71**, 3-8.
8. A. Spek, *J. Appl. Crystallogr.*, 2003, **36**, 7-13.
